# Arachidonoyl-carnitine and arachidonoyl-coenzyme A are suitable substrates for mammalian ALOX isoforms

**DOI:** 10.1016/j.jlr.2025.100861

**Published:** 2025-07-17

**Authors:** Xin Chen, Sahanawaz Parvez, Hannah F. Wiegand, Liuhui Wu, Sabine Stehling, Astrid Borchert, Junlin Yang, Polamarasetty Aparoy, Hartmut Kuhn

**Affiliations:** 1 Charité – Universitätsmedizin Berlin, Corporate Member of Freie Universität Berlin and Humboldt Universität zu Berlin, Department of Biochemistry, Berlin, Germany; 2 Molecular Modeling and Protein Engineering Lab, Biology Division, Department of Humanities and Sciences, Indian Institute of Petroleum and Energy, Visakhapatnam, Andhra Pradesh, India; 3 Department of Radiation Oncology, Cancer Center, The First Affiliated Hospital, Sun Yat-Sen University, Guangzhou, China; 4 Spine Center, Xinhua Hospital Affiliated to Shanghai Jiao Tong University School of Medicine, Shanghai, China

**Keywords:** eicosanoids, lipid peroxidation, lipoxygenases, oxidative stress, acyl carnitines, coenzyme A

## Abstract

Lipoxygenases (ALOX) convert free polyenoic fatty acids to bioactive mediators, which induce phenotypic alterations in target cells. However, the intracellular concentrations of free fatty acids are very low, as these compounds are rapidly esterified with coenzyme A. The acyl-CoA esters are subsequently used for re-acylation via the Lands cycle, or they are trans-esterified to acyl carnitines for mitochondrial import. Whether acyl carnitines and acyl-CoA derivatives might also serve as ALOX substrates has not been explored. In the present study, we prepared six different wild-type mammalian ALOX-isoforms and a selected enzyme mutant, incubated the recombinant proteins in vitro with free arachidonic acid, arachidonoyl-carnitine, and arachidonoyl-coenzyme A, and quantified the amounts of primary oxygenation products. We found that for most ALOX-isoforms, arachidonoyl-carnitine was oxygenated at a similar rate as free arachidonic acid and that the chemical structures of the primary oxygenation products were identical. In contrast, arachidonoyl-coenzyme A was oxygenated with a 3-5-fold lower rate, but here again highly specific patterns of primary oxygenation products were formed. In silico docking studies and molecular dynamics simulations suggested that free arachidonic acid and arachidonoyl-carnitine are similarly aligned at the active site of rabbit ALOX15, but the binding of arachidonoyl-coenzyme A was sterically hindered because of the bulkiness of the CoA moiety. Taken together, our data indicate that acyl carnitines and fatty acid coenzyme A esters are suitable lipoxygenase substrates and that these compounds are oxygenated to isoform-specific patterns of primary oxygenation products.

## Highlights

• ALOX isoforms oxygenate acyl carnitines and acyl coenzyme A to specific products.
• Acyl carnitines are rapidly oxygenated, but acyl CoAs are less effective substrates.
• Triad determinant mutations alter the specificity of acyl carnitine oxygenation.
• Molecular docking studies describe the structure of the enzyme–substrate complexes.

## Graphical Abstract

**Figure:**
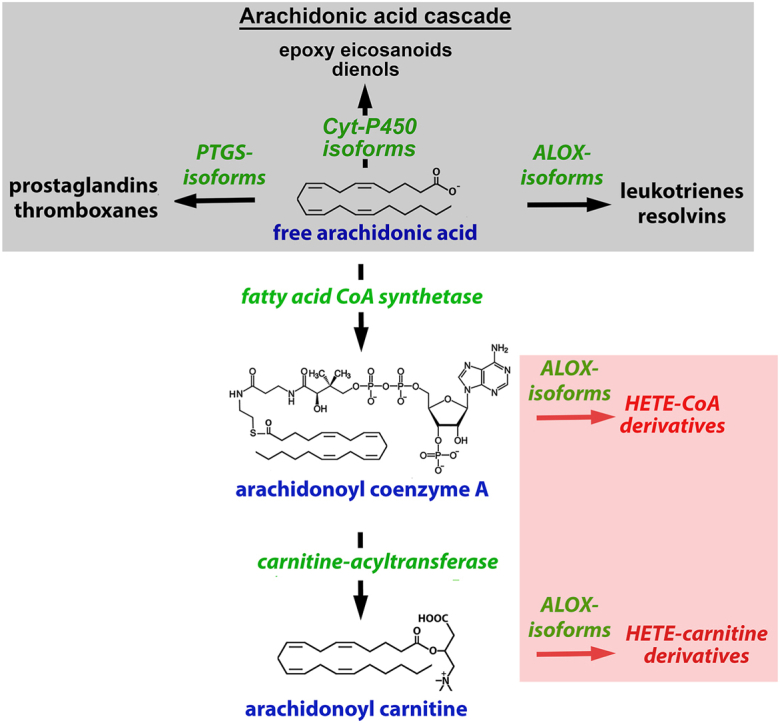

Arachidonic acid lipoxygenases (ALOX-isoforms) catalyze the oxygenation of polyenoic fatty acids (PUFAs) to lipid hydroperoxides (1, 2, 3). In the human reference genome, six functional *ALOX* genes (*ALOX5, ALOXE3, ALOX15, ALOX12, ALOX12B, ALOX15B*) have been identified (4), and experiments with isoform-specific knockout mice (5, 6, 7, 8, 9) suggested that each isoenzyme fulfills specific functions. Following the classic concept of the arachidonic acid cascade (10, 11) ALOX isoforms oxygenate free polyenoic fatty acids (PUFAs) after these substrates have been released from membrane phospholipids via the catalytic activity of ester lipid hydrolyzing enzymes, such as phospholipase A2 (12). Human ALOX5 is the key enzyme in the biosynthetic cascade of pro-inflammatory leukotrienes (13), which play important roles in the pathogenesis of allergic diseases (14), and leukotriene receptor antagonists are currently prescribed as anti-asthmatic drugs (15). ALOX12 orthologs have been implicated in the biosynthesis of hepoxilins (16), which play a role in epidermal differentiation (17) and in the pathogenesis of cardio-vascular diseases (18). Eoxins are pro-inflammatory ALOX15 metabolites (19) but this enzyme has also been implicated in the biosynthesis of anti-inflammatory and pro-resolving lipoxins (20), resolvins (21), maresins (22), and protectins (23).

However, ALOX-isoforms may also fulfill biological functions outside the arachidonic acid cascade. For instance, human ALOX5 exhibits a non-canonical activity in gene expression regulation (24). Other ALOX-isoforms such as soybean LOX1 (25), rabbit ALOX15 (26, 27), human ALOX15 (28) and human ALOX15B (29) do not only accept free PUFAs as substrate but are also capable of oxygenating phospholipids even when these substrates are incorporated in complex lipid-protein-assemblies such as biomembranes (30) and lipoproteins (31). ALOX15 catalyzed oxygenation of mitochondrial membrane lipids has been implicated in the maturational breakdown of these organelles during reticulocyte-erythrocyte transition (32, 33) and the enzyme has also been implicated in the differentiation of other cell types (34, 35). ALOX12B and ALOXE3 play important roles in skin development (36) and mutations in the *ALOX12B* gene have been related to ichthyosis (37), a genetic skin disease characterized by dry, thickened, and scaly skin (38). Alox12b knockout mice develop normally but die *post-partum* because of excessive dehydration (8). Systemic silencing of the *Aloxe3* gene induces similar but less severe phenotypic alterations (9).

The intracellular concentrations of free fatty acids are very low since these compounds induce cytotoxicity (39, 40). To avoid such effects, liberated fatty acids are rapidly esterified with coenzyme A (Fig. 1) via the catalytic activity of acyl-CoA synthetases (ACS) (41). These enzymes (42), which esterify short-chain (ACSS), medium-chain (ACSM), long-chain (ACSL), and very long-chain fatty acids (ACSVL) are evolutionary highly conserved. The human genome involves some 20 different *ACS* genes (43) and for the metabolism of polyunsaturated fatty acids, the ACSL subfamily members are of particular importance (42). These isoenzymes (ACSL1, ACSL3, ACSL4, ACSL5, ACSL6) catalyze esterification of a broad range of fatty acid substrates of different chain length, and ACSL4 prefers polyunsaturated fatty acids as substrate (44).

**Fig. 1:**
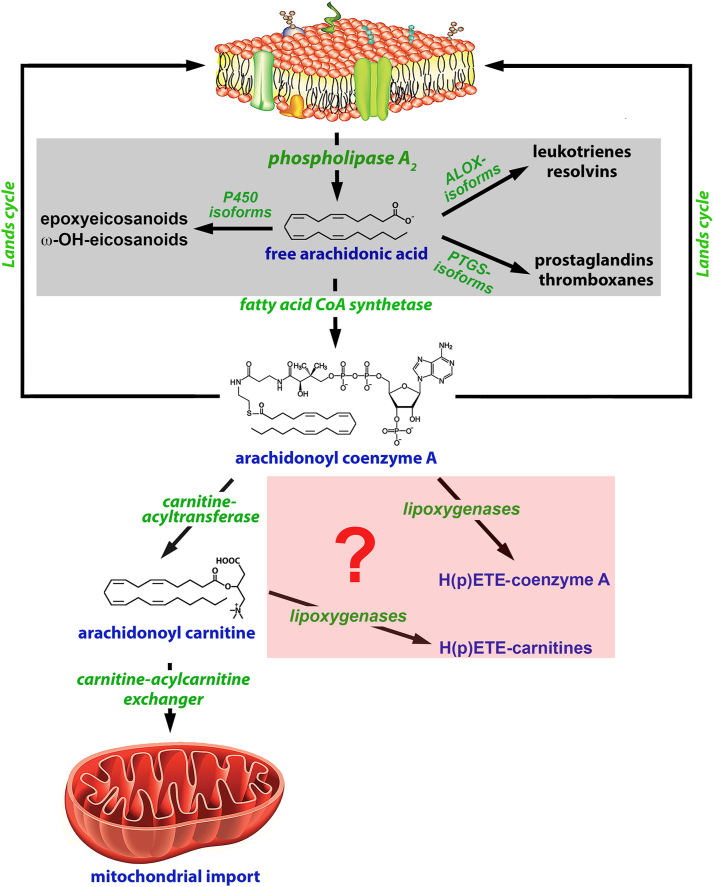
Arachidonic acid metabolism in mammalian cells. Arachidonic acid is liberated from the membrane phospholipids by the catalytic activity of phospholipase A2 and is subsequently either oxygenated by ALOX-, PTGS- and cytochrome P450 isoforms to bioactive lipid mediators (arachidonic acid cascade, grey background). Alternatively, free AA is rapidly esterified to the corresponding CoA-ester via the catalytic activity of fatty acid CoA synthetases using ATP as energy source. The resulting CoA esters may finally serve as substrate for trans-esterification into membrane phospholipids (Lands cycle) or react with carnitine to form acyl carnitines, which are subsequently imported into mitochondria to serve as substrate for mitochondrial ß-oxidation. It remains unclear whether acyl carnitines and acyl-CoA esters may serve as ALOX substrate (red shaded area) but this question was positively answered in the present study.

Acyl-CoA esters are subsequently further metabolized via two alternative metabolic routes (Fig. 1): i) Trans-esterification with lysophospholipids via the Lands cycle (45), which leads to remodeling of the membrane phospholipid bilayer. ii) Conversion to acyl-carnitines, which are subsequently imported into the mitochondria (Fig. 1), where the acyl chain of the fatty acid is broken down as an energy source via ß-oxidation. However, since AA-Car and AA-CoA carry arachidonoyl residues, these acyl esters might also serve as ALOX substrates. In fact, linoleoyl coenzyme A (LA-CoA) serves as a substrate for recombinant human ALOX15 (46), but the pattern of oxygenation products has not been explored. Other acyl-CoA derivatives such as stearoyl-CoA and oleoyl-CoA have been identified as ALOX inhibitors, and an allosteric mode of action has been suggested for these compounds (46). However, it has never been explored whether other mammalian ALOX-isoforms are also capable of oxygenating PUFA-containing CoA esters (Fig. 1, red shaded area) and whether acyl carnitines may serve as ALOX substrates. To answer these questions, we prepared different wildtype and mutant mammalian ALOX-isoforms, incubated these enzymes in vitro under identical experimental conditions with free AA, AA-Car, and AA-CoA for 3 min, quantified the formation of the primary oxygenation products, and analyzed their chemical structures, including the enantiomer composition. We found that all ALOX-isoforms were capable of oxygenating acyl-carnitines and acyl-CoA esters, but we observed isoform-specific differences in the reaction rates and the patterns of oxygenation products.

## Materials and Methods

### Chemicals

The chemicals used for this study were obtained from the following sources: Arachidonic acid (AA), linoleic acid (LA), arachidonoyl-carnitine (AA-Car), arachidonoyl-coenzyme A (AA-CoA) and authentic HPLC standards of HETE-isomers (5*S/R*-HETE, 5*S*-HETE, 8*S/R*-HETE, 8*S*-HETE, 12*S/R*-HETE, 12*S*-HETE, 15*S/R*-HETE, 15*S*-HETE) from Cayman Chem.; soybean LOX1 from Serva; acetic acid from Carl Roth GmbH; sodium borohydride from Life Technologies, Inc.; isopropyl-β-thiogalactopyranoside (IPTG) from Carl Roth GmbH; restriction enzymes from ThermoFisher; the *E. coli* strain Rosetta2 DE3 pLysS from Novagen (Merck-Millipore). HPLC-grade solvents and water were purchased from Fisher Scientific.

### Preparation of rabbit reticulocyte ALOX15

The rabbit reticulocyte ALOX15 was prepared to apparent electrophoretic homogeneity from the hemolysate of a reticulocyte-rich red blood cell suspension obtained from anemic rabbits (27). After hemolysis, the stroma-free supernatant of the hemolysate was used as the starting material, and a fractionated ammonium-sulfate precipitation was carried out. The ALOX15 was precipitated between 30% and 60% saturation. The precipitated proteins were reconstituted in 10 mM phosphate buffer (pH 7.4) and dialyzed against this buffer overnight. Finally, the enzyme was purified by anion-exchange chromatography on a semipreparative Mono Q FPLC column (Pharmacia, Uppsala, Sweden). The enzyme preparation used for these experiments showed one major band (purity degree > 95.5%) in SDS-PAGE, a molar iron load of 85%, and a molecular turnover number of linoleic acid oxygenation of 30 s^-l^ in the presence of 0.2% of sodium cholate as substrate solubilizer.

### Bacterial expression of mammalian ALOX isoforms in *E. coli*

Bacterial expression of recombinant ALOX isoforms was performed as described in (47). In brief, competent *E. coli* cells (strain Rosetta 2 DE3 pLysS) were transformed with 50–100 ng of the recombinant expression plasmids, and the cells were grown overnight on kanamycin/chloramphenicol-containing agar plates. An isolated bacterial clone was picked, and two 1 ml bacterial liquid cultures (LB medium with 50 μg/ml kanamycin and 35 μg/ml chloramphenicol) were grown for 6–8 h at 37° C under gentle agitation (180 rpm). One of these pre-cultures was then added to 50 ml of sterile culture medium (ENPRESSO B kit, Enpresso GmbH) in ultra-yield culture flasks (Thomson Instrument Company) containing kanamycin (50 μg/ml) and chloramphenicol (35 μg/ml) as antibiotics. The cells were grown overnight at 30°C and 150 rpm until the OD_600_ had reached values above 5. Expression of the recombinant proteins was then induced by the addition of Isopropyl-β-D-thiogalactopyranosid (IPTG) to reach a final concentration of 1 mM. The cultures were maintained at 22°C for 18 h at 230–250 rpm agitation. Bacteria were harvested, the resulting pellet was reconstituted in a total volume of 5 ml PBS, and bacteria were lysed by sonication (digital sonifier, W-250D Microtip Model 102, 50% maximal sonication amplitude; Branson Ultraschall). Cell debris was spun down (15 min, 15,000 *g*, 4°C), and the bacterial lysate supernatants were used for activity assays, protein quantification, SDS-PAGE, and Western blot analysis.

### Expression of human ALOX15 in Sf9 cells

Human ALOX15 is not well expressed in E. coli, and thus, we decided to express this enzyme as an N-terminal recombinant hexa-His-tag fusion protein in Sf9 insect cells. For this purpose, the ALOX15 coding region was excised from the recombinant bacterial expression plasmid and ligated into the pFastBac HT vector. Bacmids and recombinant baculovirus were generated according to the manufacturer’s instructions (Bac-to-Bac® Baculovirus Expression System, Invitrogen Life Technologies/ThermoFisher). Protein expression was initiated in *Sf9* cells (ThermoFisher) using the Insect XPRESS Medium (Biozyme Scientific GmbH) supplemented with 4 mM glutamine and 0.5% FCS. The cells were infected with the recombinant baculovirus and subsequently incubated at 27°C on an agitation platform (120–130 rpm). After 72 h of incubation (30% dead cells), the cells were harvested by centrifugation, lysed by sonication (Branson W-250P tip sonifier, Heineman), and the lysate supernatant was used as the enzyme source.

### SDS-PAGE and quantitative Western blotting

To quantify the expression of the different ALOX-isoforms in E. coli, aliquots (0.1–20 μl) of the lysis supernatants were analyzed by SDS-PAGE on a 7.5% polyacrylamide gel. Separated proteins were transferred to a nitrocellulose membrane (Thermo Scientific GmbH) by a wet blotting method (ProSieve Ex Western Blot Transfer Buffer 10x, Biozym Scientific GmbH). The membranes were blocked with blocking solution (10-fold BlueBlock PF for Blotting, SERVA Electrophoresis GmbH), washed three times with PBS containing 0.3% TWEEN 20, and were finally incubated with an anti-His-HRP antibody (Miltenyi Biotec GmbH) for 1–2 h at room temperature. After several steps of washing, the membranes were stained using the SERVALight Polaris CL HRP WB Substrate Kit (SERVA Electrophoresis GmbH) for 5 min at room temperature. Chemiluminescence was quantified using the FUJIFILM Luminescent Image Analyzer LAS-1000plus (Fujifilm Europe GmbH, Düsseldorf, Germany). For quantification of the intensity scale of the immunoblots, known amounts of pure recombinant ALOX of *Myxococcus fulvus*, which was also expressed as an N-terminal His-tag fusion protein in E. coli (48), were applied to Western blot analysis. The intensities of the immunoreactive bands were quantified using Image J software package, and from the band intensities, we calculated the amounts of ALOX proteins in the different enzyme preparations.

### In vitro ALOX activity assays

To assay the catalytic activity of the ALOX-isoforms with the different substrates, variable amounts of the enzyme preparations (pure rabbit ALOX15, bacterial lysate supernatants involving the different mammalian ALOX-isoforms) were added to 0.5 ml of PBS containing 50–100 μM of the different substrates. After a 3-minute incubation period at room temperature, the hydroperoxy derivatives formed during the incubation period were reduced to the corresponding alcohols by the addition of 1 mg of solid sodium borohydride. After 5 min on ice, the samples were acidified with 35 μl of concentrated acetic acid.

### Lipid extraction and alkaline hydrolysis

The total lipids were extracted from the acidified incubation samples following the method of Bligh and Dyer (49). For this purpose, 0.5 ml of PBS was added to the incubation samples, and the mixtures were transferred to 20 ml glass tubes. Then 2.5 ml of methanol and 1.25 ml of chloroform were added, and the samples were rigorously vortexed for 1 min (no phase separation). After 10 min on ice, 1.25 ml of chloroform and 1.25 ml of PBS were added, and this mixture was vortexed again for 1 min. For complete phase-separation, the milky-looking suspensions were centrifuged for 15 min in a Heraeus Megafuge 1.0R (Heraeus Deutschland GmbH) at 4,000 rpm. The lower phase containing the extracted lipids was recovered, 3 ml of 2-propanol was added, and the solvents were evaporated using a Heidolph rotatory evaporator (Heidolph Scientific Products GmbH). The remaining lipids were dissolved in 0.45 ml of anaerobic methanol, the samples were set under an argon atmosphere and were briefly (30 s) sonicated in a Sonorex RK512H ultrasonic bath (Brandelin Electronic GmbH). After sonication, 75 μl of anaerobic 40% KOH was added, the tubes were closed with glass stoppers, and the ester lipids were hydrolyzed for 20 min at 60° C in a dark water bath. Then the samples were cooled down on ice, 75 μl of acetic acid was added for acidification, and precipitates were removed by centrifugation. 200 μl of the clear supernatants were then injected into the RP-HPLC analysis of the oxygenation products.

### HPLC analysis of the reaction products

To quantify the oxygenation products formed during the 3 min incubation period of the enzymes with the different substrates and for preliminary structural identification of the reaction products, 200 μl of the hydrolysis mixture was injected to RP-HPLC analysis. For this purpose, a Shimadzu instrument equipped with a diode array detector (SPD-M20A) and an auto-injector (SIL-20AC) was used and metabolites were separated on a Nucleodur C18 Gravity column (Macherey-Nagel; 250 × 4 mm, 5 μm particle size) coupled with a guard column (8 × 4 mm, 5 μm particle size). A solvent system consisting of acetonitrile: water: acetic acid (70 : 30 : 0.1, by vol) was employed at a flow rate of 1 ml/min, and the analytes were eluted isocratically at 25°C. For quantification, the chromatographic scale was calibrated by injecting known amounts of 15-HETE (six-point calibration curve).

Since under our chromatographic conditions not all positional HETE isomers were baseline-resolved, and since RP-HPLC does not allow enantiomer separation, the conjugated dienes detected in RP-HPLC were recovered and further analyzed by combined normal phase/chiral-phase HPLC (NP/CP-HPLC). For this purpose, the solvents of the prepared conjugated dienes were removed with the rotatory evaporator and the remaining lipids were reconstituted in 200 μl of chiral phase HPLC column solvent (n-hexane: methonol: ethanol and acetic acid (96: 3: 1: 0.1, by vol). For combined NP/CP-HPLC a Nucleosil HPLC pre-column (4.6 × 30 mm, 5 μm particle size; Macherey-Nagel) was connected with the inlet port of a Chiralpak AD-H column (250 mm × 4.6 mm, 5 μm particle size, Daicel), and the analytes were run through the two interconnected columns. For preparation of the mobile phase, we first mixed 30 ml of methanol with 10 ml of ethanol and 1 ml of acetic acid and added this solvent mixture to 960 ml of n-hexane. It should be stressed that the mobile phase should be prepared freshly each day since long-term storage of the solvent significantly elevated the retention times of the analytes which also induces significant peak broadening. The solvent flow rate was set to 1.6 ml/min and light absorption at 235 nm (conjugated dienes) was recorded. The chemical identities of the analytes were determined by comparison of the retention times with authentic standards and by UV-spectroscopy.

### Site-directed mutagenesis and preparation of the Ile418Ala human ALOX15 mutant

In contrast to mouse Alox15, which converts free AA dominantly to 12S-HETE (5), human ALOX15 is an AA 15-lipoxygenating enzyme (28). However, the reaction specificity of human ALOX15 can easily be murinized by Ile418Ala exchange. This mutation deepens the substrate binding pocket and forces a different alignment of the substrate at the active site (50). To explore whether the reaction specificity of AA-Car oxygenation is also altered by this amino acid exchange, we prepared the enzyme mutant and compared the patterns of oxygenation products formed by wild-type human ALOX15 and its Ile418Ala mutant. Site-directed mutagenesis was carried out as described in (47) using the PfuUltra II Hotstart PCR Master Mix kit (Agilent Technologies Germany GmbH & Co. KG). In detail, 10–50 ng wild-type human ALOX15 plasmid-DNA were incubated with the mutagenesis primers (1 μl of 5 μM solution each) and 12.5 μl Pfu UltraI II Hot Start PCR Master Mix in a total volume of 25 μl adjusted with sterile water. The PCR protocol was as follows: 95°C for 1 min initial denaturation, cycle: 30 s at 95°C (denaturation phase), then 60 s at 55°C (annealing phase) followed by the synthesis phase (10 min at 68°C). This cycle was repeated 18 times. Subsequently, the parent DNA was digested with 1 μl DpnI (Thermo Scientific) for 30 min, and the digestion was concluded by incubating the samples at 80°C for 10 min 8 μl of the PCR sample was used for transformation of competent E. coli XL-1 Blue cells (Agilent Technologies Inc). After incubation for 30 min on ice, the cells were heat shocked for 45 s at 42°C, kept on ice for 2 min, and then 400 μl SOC Medium was added. After 1 h incubation at 37°C, the cells were plated on an LB-agar plate supplemented with 50 μg/ml kanamycin (for pET28b) or 100 μg/ml ampicillin (for pcDNA 3.1 or pFastBac HT) and incubated overnight at 37°C. An isolated growing clone was selected to set up a 2 ml liquid culture, plasmid DNA was prepared using the NucleoSpin Plasmid kit (Macherey & Nagel), and nucleotide sequencing (Eurofins Genomics Germany GmbH) confirmed the mutagenesis. The enzyme mutant was then expressed as described above (see Bacterial expression of mammalian ALOX isoforms in *E. coli*).

### In silico substrate docking studies and molecular dynamics simulations

To gain molecular insights into the enzyme-substrate interactions molecular docking studies were performed using the Genetic Optimization for Ligand Docking (GOLD) software. The crystal structure of rabbit reticulocyte 15S-lipoxygenase [PDB ID: 2P0M, (51)] was used as receptor for the docking studies. Ligand structures were retrieved from the PubChem database (https://pubchem.ncbi.nlm.nih.gov), and their geometries were optimized using GAUSSIAN 16 software (52). Geometry optimization was carried out using the B3LYP functional with the 6-31G basis set to obtain energy-minimized conformers. The optimized ligand structures were subsequently used for docking. Protein preparation was conducted using BIOVIA Discovery Studio Visualizer. Specifically, chain B, which was co-crystallized with the native inhibitor, was selected for the docking studies. All crystallographic water molecules were removed to avoid unwanted interactions. The monomeric form of the protein (chain B only), along with the native ligand, was employed for docking studies. Hydrogen atoms were added to the protein structure using Hermes Visualizer. A 10 Å spherical grid was generated around the centroid coordinates of the ligand in the crystal structure, encompassing the non-heme iron atom, a critical component of the active site. Ligand molecules were docked using the default parameters in GOLD (53). A total of 100 genetic algorithm (GA) runs were performed for each ligand to exhaustively explore possible conformational flexibilities within the binding site. Molecular docking was carried out using three specific constraints to better reflect the biological relevance of ligand binding. The first constraint involved defining Arg403 as a potential hydrogen bond donor, guiding the orientation of the ligand’s polar functional groups (54). The second constraint enforced a 3 Å distance between the C13 atom of the ligand’s aliphatic chain and the non-heme iron atom at the active site and third, the methyl terminus of AA-car was placed to remain within 3 Å of the side chain of Ile418. The resulting docking poses were further analyzed and visualized using BIOVIA Discovery Studio Visualizer to assess interactions and binding orientations within the active site.

To optimize the enzyme-substrate complexes, we conducted Molecular Dynamics (MD) simulations using the high-performance molecular dynamics engine Desmond (2025-1) for the enzyme–substrate complexes (55). For this purpose, the enzyme protein was solvated in an orthorhombic box and neutralized with 0.15 M NaCl. Energy minimization was performed for 250 ps to eliminate unfavorable contacts. The system was then equilibrated under an isothermal-isobaric ensemble that was followed by a 100 ns production MD simulation.

### Sample repetitions, statistical evaluation, and image preparation

If not indicated otherwise, 4–5 repetitions were run for each sample. The area units of the HPLC peaks were converted into nmol conjugated dienes after calibration of the chromatographic system (injection of six different amounts of 15-HETE). Statistical evaluation of the experimental raw data and quantification of the patterns of AA oxygenation products was carried out with the Mann-Whitney U-test using the GraphPad Prism software package (version 8.3.1). Numeric *P*-values <0.05 were considered statistically significant. The images were prepared using the GraphPad Prism software (version 8.3.1) and the Adobe Photoshop program (version 21.1.1).

## Results

### Pure rabbit ALOX15 oxygenates AA-carnitine at a higher rate as free AA, but AA-CoA is a less efficient substrate

Polyunsaturated fatty acid (PUFA) derivatives carrying an esterified carboxylic group, such as AA methyl ester, are less efficient substrates for different ALOX-isoforms when compared with free AA (56). On the other hand, anandamide and 2-arachidonoyl glycerol, in which the carboxylic group was also modified, are better substrates for mammalian ALOX-isoforms (57). Unfortunately, it has never been explored whether AA-carnitine and AA-coenzyme A esters, which do not carry a free carboxylate, are suitable substrates for these enzymes.

**Fig. 2:**
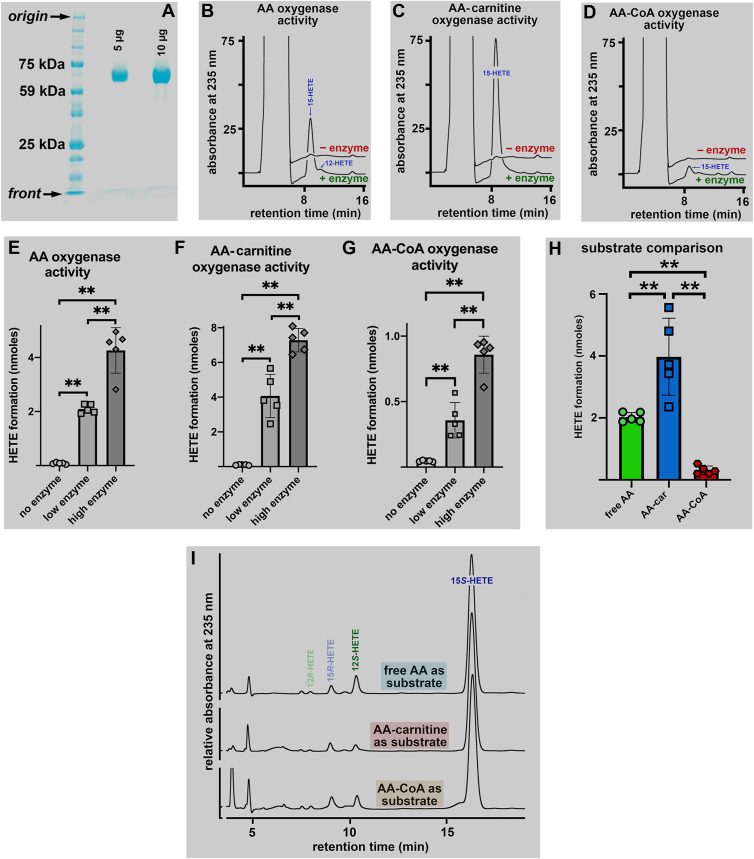
Oxygenation of free arachidonic acid, arachidonoyl-carnitine and arachidonoyl-coenzyme A by pure rabbit ALOX15. 3 and 30 μl (2 mg/ml ALOX protein) of a solution of pure rabbit ALOX15 were incubated for 3 min at room temperature in 0.5 ml PBS containing the three different substrates (free AA, AA-Car, AA-CoA) at a concentration of 50 μM. For the “no enzyme” control incubations 30 μl of PBS was used as enzyme equivalent. The reaction was terminated by the addition of 1 mg of solid sodium borohydride. Sample work-up including lipid extraction and alkaline hydrolysis of the ester lipids as well as HPLC analysis of the reaction products are described in Materials and Methods. A: SDS-PAGE of the final rabbit ALOX15 preparation. B: RP-HPLC of conjugated dienes formed from free AA. C: RP-HPLC of conjugated dienes formed from AA-Car. D: RP-HPLC of conjugated dienes formed from AA-CoA. E: Statistical evaluation of the AA oxygenase activities (no enzyme, 30 μl PBS; low enzyme, 3 μl rabbit ALOX15 preparation; high enzyme, 30 μl rabbit ALOX15 preparation), n = 5. F: Statistical evaluation of the AA-Car oxygenase activities (no enzyme, 30 μl PBS; low enzyme, 3 μl rabbit ALOX15 preparation; high enzyme, 30 μl rabbit ALOX15 preparation), n = 5. G: Statistical evaluation of the AA-CoA oxygenase activities (no enzyme, 30 μl PBS; low enzyme, 3 μl rabbit ALOX15 preparation; high enzyme, 30 μl rabbit ALOX15 preparation), n = 5. (H) Comparison of the oxygenation rates of the three different substrates (3 μl enzyme preparations, n = 5). (I) NP/CP-HPLC analyses of the reaction products formed from free AA (upper trace), AA-Car (middle trace) and AA-CoA (lower trace). Mann-Whitney U-test, ∗∗*P* < 0.01.

To answer this question, we first prepared pure rabbit ALOX15 from a reticulocyte-rich red blood cell suspension of rabbits. The final preparation was pure (>95%) in SDS-PAGE (Fig. 2A). Next, we incubated this enzyme in side-by-side experiments with free arachidonic acid (AA), arachidonoyl-carnitine (AA-Car), and arachidonoyl-coenzyme A (AA-CoA) for 3 min, hydrolyzed the lipid extracts, and quantified by RP-HPLC the amounts of primary oxygenation products formed during the incubation period (Fig. 2B–D). Here we found (Fig. 2B) that free AA was oxygenated to conjugated dienes co-eluting with an authentic standard of 15-HETE. Smaller amounts of 12-HETE were also formed, and these data confirm previous results of the reaction specificity of this enzyme with AA (28). When AA-Car was used as substrate, higher amounts of 15- and 12-HETE were detected (Fig. 2C). Thus, AA-Car was oxygenated by rabbit ALOX15 at a 2-fold higher rate. In contrast, when AA-CoA was used as substrate, we only detected small amounts of conjugated dienes (Fig. 2D). However, when compared with the no-enzyme control incubations, we clearly detected oxygenation products. Thermal inactivation of the enzyme (5 min at 90°C) completely abolished the formation of conjugated dienes (supplemental Fig. S1A–D), which indicated the enzymatic origin of the conjugated dienes. Taken together, this data indicates that AA-CoA was also a substrate for rabbit ALOX15, but its oxygenation rate was lower than that of free AA oxygenation.

To put this conclusion on a broader experimental basis, we repeated the incubations using ten-fold higher enzyme concentrations (Fig. 2E–G). Here, we confirmed that free AA, AA-Car, and AA-CoA were suitable substrates for rabbit ALOX15. However, evaluating the data, we noticed that a 10-fold increase in the enzyme concentrations only induced a 2- to 3-fold increase in conjugated diene formation. To explain this finding, we analyzed the disappearance of the substrate during the incubation period and found that at high enzyme concentrations, more than 90% of the substrates were consumed, and this was the case for all substrates (supplemental Fig. S2A–I).

To confirm the substrate ranking (AA-Car > free AA > AA-CoA) suggested in Figure 2B–D, we statistically evaluated all activity data (Fig. 2H) and found that under our experimental conditions, AA-Car was a two-fold better substrate than free AA. In contrast, AA-CoA was 5-fold less effective. It should be stressed at this point that this conclusion was not based on the results of detailed kinetic experiments. This is clearly a limitation of the present study, and we will discuss this point later on in this article (Limitation section).

Since our RP-HPLC system does not well resolve all HETE-isomers (12-HETE and 8-HETE are not resolved) and since HETE enantiomers are not at all separated, we prepared the conjugated dienes formed from the three substrates by RP-HPLC and re-analyzed them by combined normal phase/chiral phase HPLC (Fig. 2I). Here, we found a strong preponderance of 15S-HETE over the corresponding R-enantiomers. This high degree of enantio-selectivity indicated that the oxygenation reaction with all substrates was tightly controlled by the enzyme. When we analyzed the free AA oxygenation products, we also detected significant amounts of 12S-HETE (10% of the conjugated dienes), which is consistent with previous findings (58, 59). For AA-Car and AA-CoA, the share of 12S-HETE formation is somewhat reduced, and these data suggest subtle differences in the substrate alignment at the active site of the enzyme.

### AA-carnitine and AA-CoA ester are also suitable substrates for other mammalian ALOX-isoforms

To explore whether other mammalian ALOX-isoforms are also capable of oxygenating AA-Car and AA-CoA, we expressed different human and mouse ALOX-isoforms as recombinant N-terminal His-tag fusion proteins and used crude enzyme preparations (cellular lysate supernatants) as enzyme source. Since the epidermal ALOX-isoforms (ALOX12B, ALOXE3) are difficult to express and since mouse and human ALOX5 are not capable of oxygenating modified AA derivatives (57) we selected human and mouse ALOX15, human and mouse Alox15b, and human ALOX12 as candidate enzymes. Since human and mouse ALOX15 orthologs, on the one hand, and human and mouse ALOX15B orthologs on the other, exhibit different reaction specificities with free AA, it was interesting to explore whether similar differences could also be detected with AA-car and AA-CoA. Applying our bacterial expression strategy (see Materials and Methods) we found that except for human ALOX15, all enzymes were well expressed in E. coli and could be detected in the bacterial lysate supernatant. In contrast, human ALOX15 was mainly localized as a catalytically inactive protein in the pellet of the bacterial lysate. Thus, we were forced to express this enzyme in Sf9 insect cells. The expression efficiency (quantitative Western blotting) of the different enzymes is shown in Table 1.

**Table 1:** Recombinant expression of mammalian ALOX-isoforms in pro- and eukaryotic expression systems

Enzyme	Expression System	Expression Level (mg/l Liquid Culture)
human ALOX15	*Sf9 cells*	96
mouse Alox15	*E. coli*	200
human ALOX15B	*E. coli*	55
mouse Alox15b	*E. coli*	89
human ALOX12	*E. coli*	350

The different mammalian ALOX-isoforms were expressed as recombinant N-terminal His-tag fusion proteins and quantitative Western-blot analyses (see Materials and Methods) were carried out to quantify the expression levels of the different proteins.

Next, we attempted to purify the recombinant proteins by affinity chromatography on Ni-agarose. Although these attempts were successful for human ALOX15, human ALOX15B, and human ALOX12, we failed to recover a catalytically active protein for mouse Alox15 and mouse Alox15b. Thus, we decided to employ crude enzyme preparations (cellular lysate supernatants) for our comparative experiments.

**Fig. 3:**
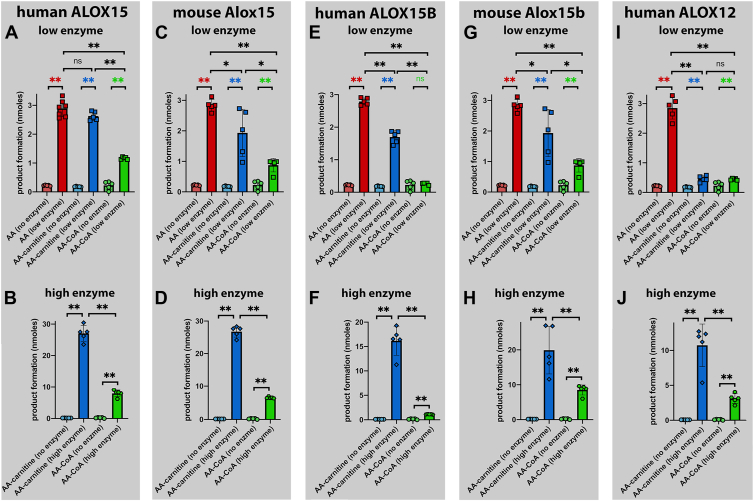
Oxygenation of free arachidonic acid, arachidonoyl-carnitine and arachidonoyl-coenzyme A by different mammalian ALOX-isoforms. Variable volumes of the different enzyme preparations, which corresponded to a similar AA oxygenase activity, were incubated for 3 min at room temperature in 0.5 ml PBS containing the three different substrates (free AA, AA-Car, AA-CoA) at a concentration of 100 μM. The reaction was terminated by the addition of 1 mg of solid sodium borohydride. Sample work-up and HPLC analysis of the reaction products are described in Materials and Methods. A: Human ALOX15 at low enzyme concentrations. B: Human ALOX15 at 10-fold higher enzyme concentrations. C: Mouse Alox15 at low enzyme concentrations. D: Mouse Alox15 at 10-fold higher enzyme concentrations. E: Human ALOX15B at low enzyme concentrations. F: Human ALOX15B at 10-fold higher enzyme concentrations. (G) Mouse Alox15b at low enzyme concentrations. (H) Mouse Alox15b at 10-fold higher enzyme concentrations. (I) Human ALOX12 at low enzyme concentrations. (J) Human ALOX12 at 10-fold higher enzyme concentrations. n = 5–7; ns, not significant, ∗*P* < 0.05, ∗∗*P* < 0.01, Mann-Whitney U-test.

To test the catalytic activities of these enzymes with the three different substrates (free AA, AA-Car, AA-CoA), we carried out in vitro activity assays (Materials and Methods) using a substrate concentration of 100 μM. As indicated in Figure 3A, human ALOX15 oxygenated AA-Car with similar efficiency as free AA, but AA-CoA was a less suitable substrate. In the no-enzyme control incubations, we only detected small amounts of oxygenation products. In fact, these products are already present in our substrate stock solutions.

Next, we repeated the experiments at 10-fold higher enzyme concentrations and observed a similar difference in the reaction rates of AA-Car and AA-CoA oxygenation. Here again, AA-Car was a better substrate than AA-CoA (Fig. 3B). For mouse Alox15, the situation was similar. Although we found that the free AA oxygenase activity of this enzyme was statistically higher than the AA-Car oxygenase activity, the rates were rather similar (Fig. 3C). The oxygenation rate of AA-CoA was somewhat lower, and thus the following substrate ranking was observed: free AA > AA-Car > AA-CoA. The oxygenation rate of AA-CoA was only 30% of that of free AA. When we repeated the experiment at higher enzyme concentrations (Fig. 3D), we confirmed that AA-Car was a 4-fold better substrate than AA-CoA.

A similar situation was observed for human ALOX15B. Free AA was a better substrate than AA-Car, but at low enzyme concentrations, we did not detect an AA-CoA activity (Fig. 3E). In fact, there was no significant difference between the no-enzyme control incubation and the ALOX15B sample. However, when we performed the experiment at 10-fold higher enzyme concentrations (Fig. 3F), we convincingly showed that this enzyme also exhibited a small but statistically significant AA-CoA oxygenase activity. In this experiment, we also confirmed that AA-Car was a better substrate for human ALOX15B than AA-CoA (Fig. 3F). For mouse Alox15b, a similar substrate behavior was observed, and the following substrate ranking was established: free AA > AA-Car > AA-CoA (Fig. 3G). At higher enzyme concentrations (Fig. 3H), we confirmed that AA-Car was a better substrate than AA-CoA.

Finally, we employed human ALOX12 as a catalyst, and here we found that the oxygenation rates of AA-Car and AA-CoA were much lower than those of free AA (Fig. 3I). However, the product content in the no-enzyme control incubations was significantly lower than that in the enzyme incubations, and these data indicate that human ALOX12 also exhibits significant AA-Car and AA-CoA oxygenase activities. This conclusion was confirmed at 10-fold higher enzyme concentrations (Fig. 3J).

**Fig. 4:**
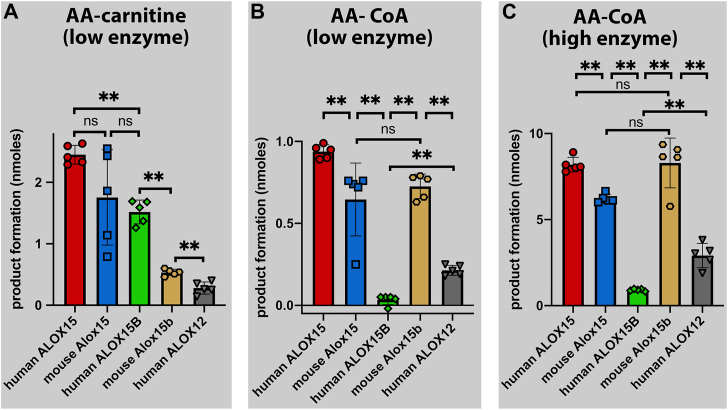
Comparison of the arachidonoyl-carnitine and the arachidonoyl-coenzyme A oxygenase activities of different mammalian ALOX-isoforms. Variable volumes of the different enzyme preparations, which correspond to the same AA oxygenase activity, were incubated for 3 min at room temperature in 0.5 ml PBS containing the two different substrates (free AA, AA-Car, AA-CoA) at a concentration of 100 μM. The reaction was terminated by the addition of 1 mg of solid sodium borohydride. Sample work-up and HPLC analysis of the reaction products are described in Materials and Methods. A: AA-Car as ALOX substrate at low enzyme concentration. B: AA-CoA as ALOX substrate at low enzyme concentration. C: AA-CoA as ALOX substrate at 10-fold higher enzyme concentrations. n = 5; ns, not significant, ∗*P* < 0.05, ∗∗*P* < 0.01, Mann-Whitney U-test.

Next, we compared the AA-Car and AA-CoA oxygenase activities of the different enzymes. For this purpose, we first subtracted the means of the conjugated dienes present in the no-enzyme control incubations from the individual values of the enzyme incubations and then we compared these differences for the five enzymes preparation. As indicated in Figure 4A all enzymes tested exhibited an AA-Car oxygenase activity since the differences between the no-enzyme control incubations and enzyme catalysis were always positive. Statistic evaluation of the experimental raw data suggested that when normalized to a similar AA oxygenase activity human ALOX15, mouse Alox15 and human ALOX15B exhibited similar AA-Car oxygenase activities. In contrast, the AA-Car oxygenase activities of mouse Alox15b and human ALOX12 were somewhat lower.

A different enzyme preference was observed when AA-CoA was used as substrate (Fig. 4B). Here, human ALOX15 was the best enzyme, and the following enzyme ranking resulted: human ALOX15 > mouse Alox15 = mouse Alox15b > human ALOX12 > human ALOX15B. This ranking was confirmed at 10-fold higher enzyme concentrations (Fig. 4C). It should be stressed at this point that our substrate rankings for the different ALOX-isoforms were not based on detailed kinetic studies. For most experiments, crude enzyme preparations (bacterial lysate supernatants) were used, and thus, no reliable kcat values could be determined. In other words, the catalytic efficiencies of the various ALOX-isoforms (k_cat_/K_M_) with the three different substrates (free AA, AA-car, AA-CoA) have not been quantified.

### Mouse Alox15b and human ALOX12 form different patterns of oxygenation products from free arachidonic acid and arachidonoyl-carnitine

**Fig. 5:**
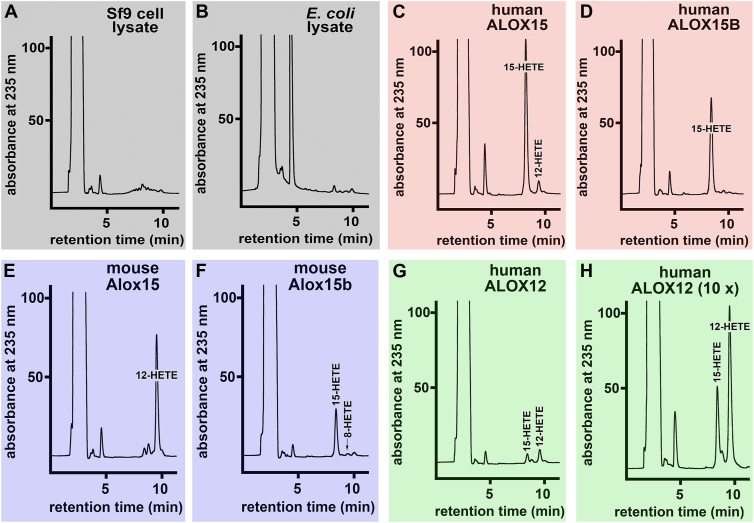
RP-HPLC analysis of the primary arachidonoyl-carnitine oxygenation products formed by recombinant mammalian ALOX-isoforms. Different volumes of the different enzyme preparations corresponding to the same AA oxygenase activity were incubated for 3 min at room temperature in 0.5 ml PBS containing AA-Car as ALOX substrate at a concentration of 100 μM. The reaction was terminated by the addition of 1 mg of solid sodium borohydride. Sample work-up and HPLC analysis of the reaction products are described in Materials and Methods. A: A cellular lysate supernatant of Sf9 cell, which were infected with a non-recombinant baculovirus, was used as enzyme source (Sf9 control). B: A cellular lysate of E. coli cells, which were transformed with a non-recombinant expression plasmid, was used as enzyme source. C: Recombinant human ALOX15 expressed in Sf9 cells was used as enzyme source. D: Recombinant human ALOX15B expressed in E. coli was used as enzyme. E: Recombinant mouse Alox15 expressed in E. coli was used as enzyme. F: Recombinant mouse Alox15b expressed in E. coli was used as enzyme. G: Recombinant human ALOX12 expressed in E. coli was used as enzyme. H: Recombinant human ALOX12 expressed in E. coli was used as enzyme (10-fold higher enzyme concentrations as compared with panel G). The retention times of authentic standards are indicated above the traces.

When cellular lysates of Sf9 cells, which were infected with a non-recombinant baculovirus, were incubated in vitro with AA-Car, no conjugated dienes were observed (Fig. 5A). Similar results were obtained when a lysate supernatant of E. coli cells was used, which were transformed with a non-recombinant expression plasmid (Fig. 5B). Recombinant human ALOX15 exhibits a dual reaction specificity with free AA. 15-HETE (90%) and 12-HETE (10%) were the major oxygenation products (28). When this enzyme oxygenated AA-Car, a similar product pattern was observed (Fig. 5C). Human ALOX15B oxygenates free AA almost exclusively to 15-HETE (60), and here we found that this was also the case for AA-Car (Fig. 5D). Mouse Alox15 oxygenates free AA predominantly to 12-HETE, which is consistent with previous findings (5), and this compound was also identified as a major AA-Car oxygenation product (Fig. 5E).

Mouse Alox15b oxygenates free AA almost exclusively to 8S-HETE (61). Here, we observed that AA-Car was almost exclusively oxygenated to 15-HETE (Fig. 5F), and these data contradict previous findings on the reaction specificity of this enzyme with free AA (61). However, our findings are consistent with the results of more recent experiments, in which AA-containing phospholipids were provided as substrate in the form of nanodiscs (29). Human ALOX12, which oxygenated free AA exclusively to 12-HETE (62), did not effectively oxygenate AA-Car (Fig. 5G), and the amounts of oxygenation products formed during our comparative studies were not sufficient for detailed structural analysis. Thus, we carried out additional activity assays using 10-fold higher enzyme concentrations. Here, we found enough conjugated dienes. Although 12-HETE was the major AA-Car oxygenation product, large amounts of 15-HETE were also formed (Fig. 5G + H). Taken together, our results suggest that free AA and AA-Car are similarly aligned with the active site of human ALOX15, human ALOX15B, and mouse Alox15. However, a different substrate alignment might be predicted for mouse Alox15b and human ALOX12.

**Fig. 6:**
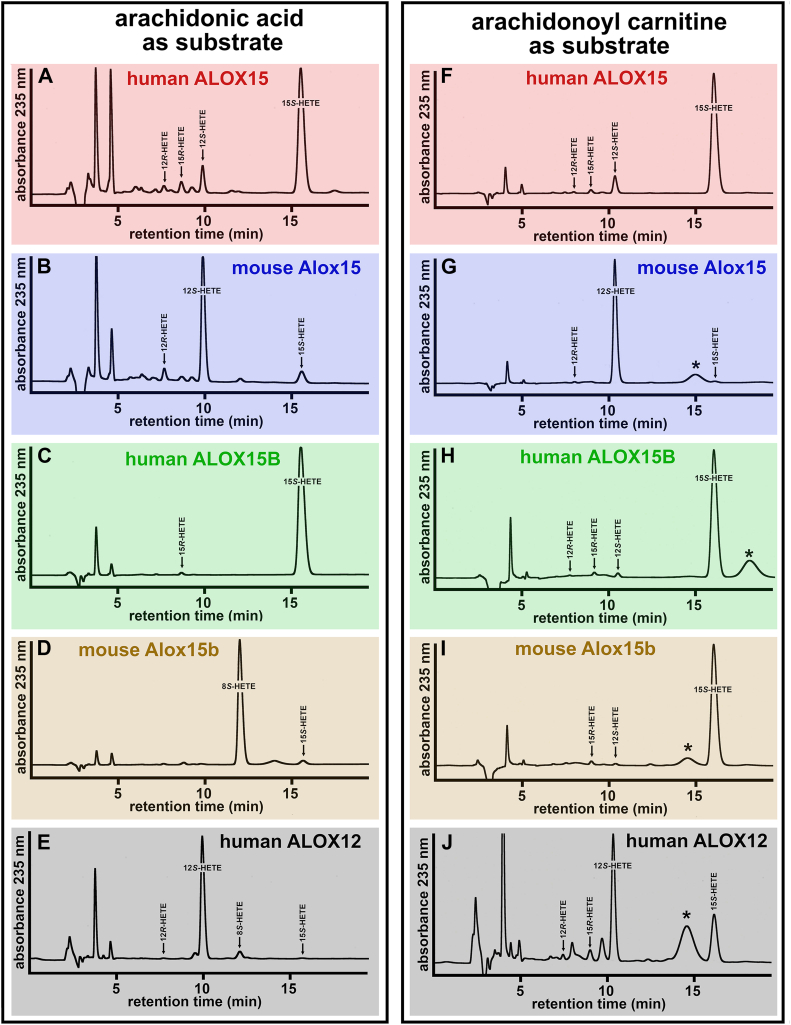
Combined NP/CP-HPLC analysis of the reaction products formed by different mammalian ALOX-isoforms from free arachidonic acid (AA) and arachidonoyl carnitine (AA-Car). In vitro activity assays and sample work-up were carried out as described in the legend to Figure 4. Conjugated dienes were prepared by RP-HPLC and subsequently analyzed by combined NP/CP-HPLC (see Materials and Methods). A: Human ALOX15 with free AA as substrate. B: Mouse Alox15 with free AA as substrate. C: Human ALOX15B with free AA as substrate. D: Mouse Alox15b with free AA as substrate. E: Human ALOX12 with free AA as substrate. F: Human ALOX15 with AA-Car as substrate. G: Mouse Alox15 with AA-Car as substrate. H: Human ALOX15B with AA-Car as substrate. I: Mouse Alox15b with AA-Car as substrate. J: Human ALOX12 with AA-Car as substrate. The retention times of authentic standards are indicated above the traces. Peaks labeled with ∗ originate from the previous HPLC run.

To explore the enantiomer composition of the oxygenation products, we prepared the conjugated dienes formed during the activity assays by RP-HPLC and analyzed them by combined normal phase/chiral phase (NP/CP) HPLC (see Materials and Methods for methodological details). Here we confirmed that human ALOX15 converted AA mainly to 15S- and 12S-HETE (Fig. 6A). The corresponding R-enantiomers were only present in small amounts, and this was also the case for AA-Car (Fig. 6F). The enantio-selectivity of mouse Alox15 was also high since 12S-HETE was the major oxygenation product of both free AA and AA-Car (Fig. 6B + G).

Human ALOX15B oxygenates AA almost exclusively to 15S-HETE (60) and here we confirmed this finding (Fig. 6C). When AA-Car was used as substrate, the product pattern was very similar, since according to our data, 15S-HETE was the dominant AA-Car oxygenation product (Fig. 6H). Mouse Alox15b converts free AA almost exclusively to 8S-HETE (61, 63) and we confirmed this finding (Fig. 6D). However, with AA-Car, 15S-HETE was the dominant oxygenation product, and the enantio-selectivity was > 95% (Fig. 6I). These data indicate that AA-Car oxygenation was tightly controlled by the enzyme. Human ALOX12 oxygenated free AA almost exclusively to 12S-HETE (Fig. 5E), but when AA-Car was used as substrate, 15-HETE was also formed (Fig. 6H). For both products, the S-enantiomers dominated (Fig. 6J). Taken together, our data on the composition of the AA-Car oxygenation products strongly suggest that the stereochemistry of the reaction is tightly enzyme-controlled. The radical reaction intermediates remained enzyme-bound and did not escape from the active side of the enzyme.

### The product patterns formed from arachidonoyl-coenzyme A were different from those of free arachidonic acid and arachidonoyl-carnitine

Compared with free AA and AA-Car, arachidonoyl-coenzyme A (AA-CoA) was a less suitable substrate for mammalian ALOX isoforms (Fig. 3A–J), and thus, we used 10-fold higher enzyme concentrations to analyze the structure of reaction products.

**Fig. 7:**
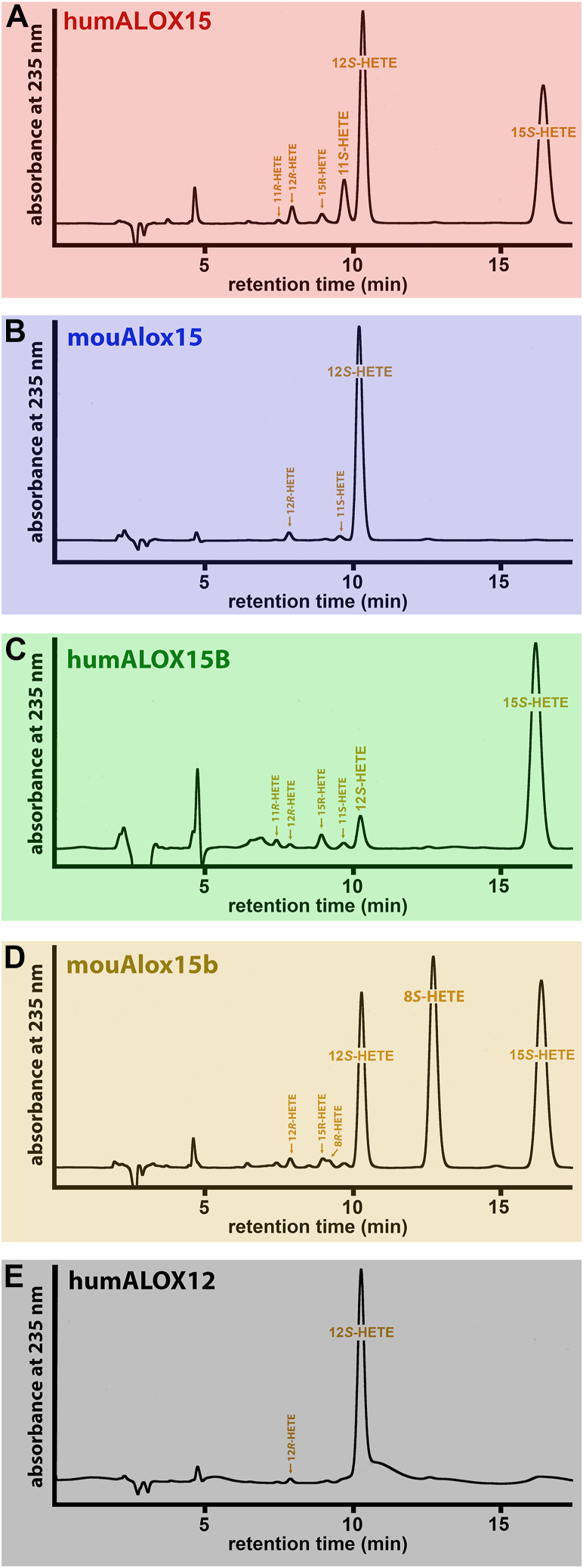
Combined NP/CP-HPLC analysis of the reaction products formed by different mammalian ALOX-isoforms from arachidonoyl-coenzyme A (AA-CoA). In vitro activity assays were carried out as described in the legend to Figure 3 and high enzyme concentrations were employed. Conjugated dienes were prepared by RP-HPLC and subsequently analyzed by combined NP/CP-HPLC (see Materials and Methods). A: Human ALOX15. B: Mouse Alox15. C: Human ALOX15B. D: Mouse Alox15b. E: Human ALOX12. The retention times of authentic standards are indicated above the traces.

Human ALOX15 oxygenated free AA and AA-Car to a 10:1 mixture of 15S- and 12S-HETE (Fig. 6A + F). Interestingly, when oxygenating AA-CoA, 12S-, and 15S-HETE were also formed in a similar ratio, but smaller amounts of 11S-HETE were also detected (Fig. 7A). The corresponding R-enantiomers (12R-HETE, 15R-HETE, and 11R-HETE) were only present at lower quantities, and these data indicate a high degree of enantioselectivity of the oxygenation reaction. Mouse Alox15 converted free AA and AA-Car mainly to 12S-HETE (Fig. 6B + G), and the same compounds was identified as major AA-CoA oxygenation product (Fig. 7B). Interestingly, we did not detect any 15S-HETE, which was present in the product mixture formed from free AA (Fig. 6B). Human ALOX15B converted free AA exclusively to 15S-HETE (Fig. 6C). With AA-CoA as substrate 15S-HETE was also detected as major oxygenation product but here about 10% of 12S-HETE were also observed (Fig. 7C). The corresponding R-enantiomers were only present in small amounts.

Mouse Alox15b effectively oxygenated AA-CoA (Fig. 4B + C). This enzyme converted free AA almost exclusively to 8S-HETE, but with AA-Car as substrate, 15S-HETE was dominant (Fig. 6D + I). With AA-CoA as substrate, this enzyme produced a complex mixture of chiral oxygenation products. As indicated in Fig. 7D, similar amounts of 15S-HETE, 12S-HETE, and 8S-HETE were formed, but the corresponding R-enantiomers were lacking. The formation of 12S- and 8S-HETE involves initial hydrogen abstraction from C10 of the arachidonic acid backbone and most probably an inverse substrate orientation at the active site. 15S-HETE formation involves C13 hydrogen abstraction, but inverse substrate alignment would force 11-HETE formation. However, we observed 11S- nor 11R-HETE among the major oxygenation products and thus, C13 hydrogen abstraction from inversely oriented substrate is obviously not catalyzed.

Human ALOX12 exhibited only a low AA-CoA oxygenase activity (Fig. 3I + J). However, using high enzyme concentrations, we detected the specific formation of 12S-HETE (Fig. 7E). Other HETE isomers could not be detected, and thus, despite the low reaction rate, the stereo-control of the oxygenation reaction was surprisingly high.

### Mutagenesis of the triad determinants in mammalian ALOX15 orthologs alters the reaction specificity of free AA and AA-car oxygenation in a similar way

In mammals, two types of ALOX15 orthologs can be differentiated, and most mammals express AA-12-lipoxygenating enzymes (47). In contrast, highly developed hominids, including extant and extinct human subspecies, express AA-15-lipoxygenating enzymes (47). According to the Triad concept (64), the reaction specificity of mammalian ALOX15 orthologs with free AA can be predicted from the amino acid sequence of these enzymes. Ile418Ala exchange in human ALOX15 converts the AA-15-lipoxygenating protein into an AA 12-lipoxygenating enzyme (47). In other words, Ile418Ala exchange murinized the reaction specificity of human ALOX15. On the other hand, Leu353Phe exchange converted the AA 12-lipoxygenating mouse Alox15 to an AA 15-lipoxygenating enzyme and thus, this point mutation humanized the reaction specificity of the mouse enzyme (65). This concept not only works with recombinant proteins but also under in vivo conditions. In fact, peritoneal macrophages of Alox15 knock-in mice, which express the Leu353Phe mutant Alox15 instead of the wild-type enzyme, convert AA predominantly to 15S-HETE (65). As a structural basis for the observed specificity differences, an altered substrate alignment at the active site of the mutant enzymes (Ile418Ala human ALOX15, Leu353Phe mouse Alox15) has been suggested.

**Fig. 8:**
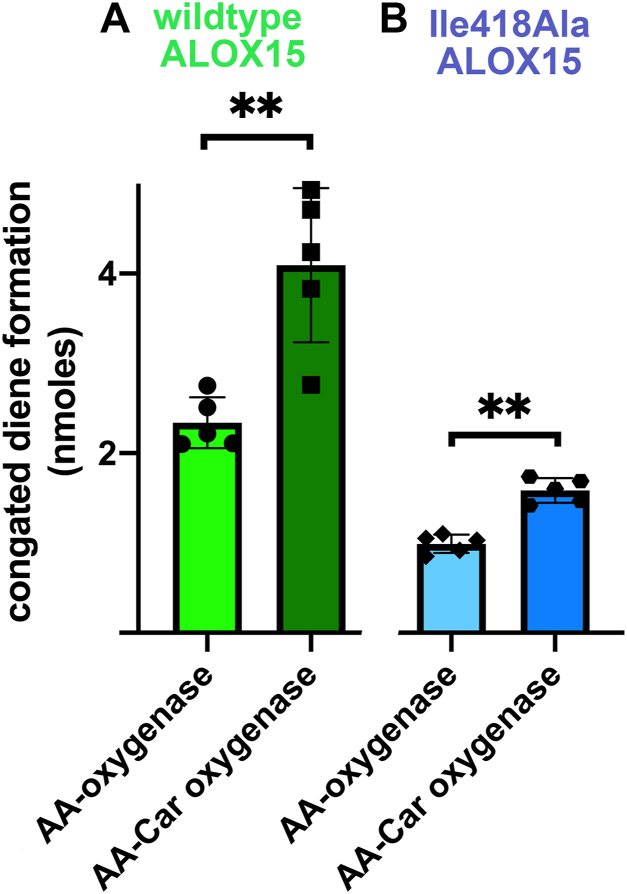
AA-oxygenase and AA-Car oxygenase activities of recombinant wildtype rabbit ALOX15 and its Ile418Ala mutant. Wild-type and mutant rabbit ALOX15 were expressed as recombinant N-terminal His-tag fusion proteins in E. coli (see Materials and Methods) and bacterial lysate supernatants were used as enzyme source. In vitro activity assays were carried out with the two substrates and the amounts of conjugated dienes formed during the 3 min incubation period were quantified. A: Wildtype enzyme, (B) Ile419Ala mutant. The mutant enzyme was expressed at lower levels, which explains the lower AA oxygenase activity of this enzyme preparation. Mann–Whitney *U* test, n = 5, ∗∗*P* < 0.01.

To explore whether a similar change in the reaction specificity can be observed when AA-Car is used as substrate, we first incubated wild-type rabbit ALOX15 and its Ile418Ala mutant with both free AA and AA-Car and analyzed the hydrolyzed lipid extracts for the presence of HETE isomers. When we compared the amounts of conjugated dienes formed during the incubation period, we found that for the wild-type enzyme, the AA-Car oxygenase activity was about 2-fold higher than that of free AA oxygenation (Fig. 8A), and these data were consistent with the results obtained for the purified enzyme (Fig. 2H). For the I418A mutant (Fig. 8B), we obtained similar data. Here again, the AA-Car oxygenase activity was significantly higher than the free AA-oxygenase activity.

Next, we analyzed the reaction products formed by the two enzyme variants from the two different substrates. Since our RP-HPLC system does not properly resolve all HETE-isomers (12-HETE and 8-HETE are not well resolved) and since HETE enantiomers cannot be resolved in RP-HPLC we prepared the conjugated dienes by RP-HPLC and subsequently analyzed them by combined normal phase/chiral phase HPLC.

**Fig. 9:**
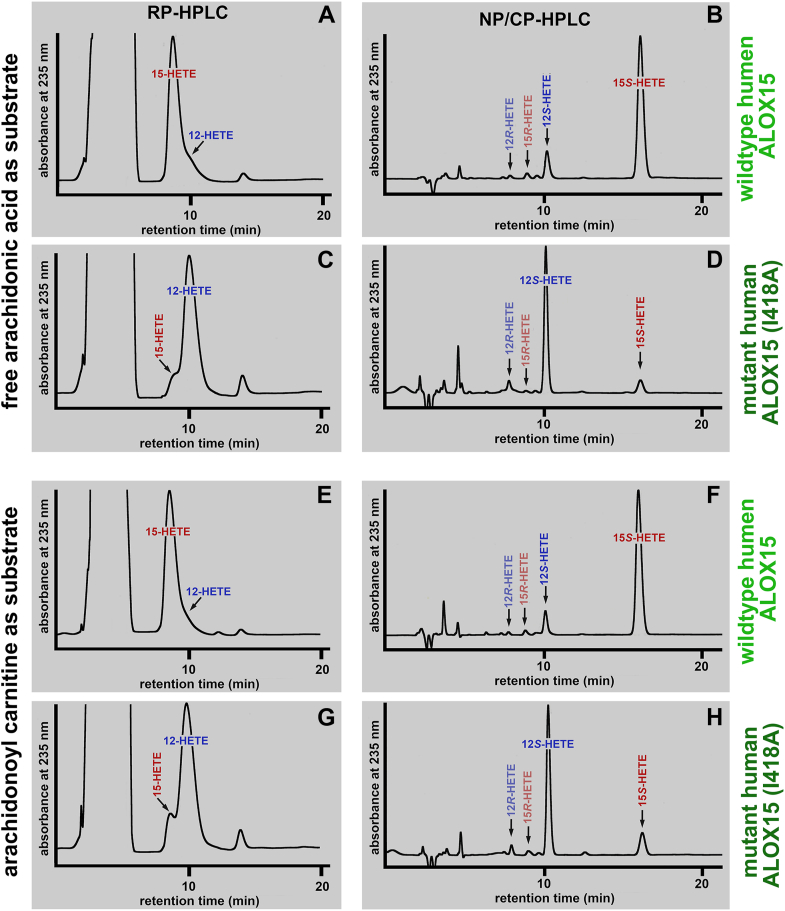
Product patterns formed from free AA and AA-Car by wildtype rabbit ALOX15 and its Ile418Ala mutant. The reaction products formed from free AA and AA-Car (see legend to Fig. 8) were prepared by RP-HPLC (panels A, C, E, G) and further analyzed by combined NP/CP-HPLC (panels B, D, F, H).

As expected, free AA is oxygenated by wildtype human ALOX15 predominantly to 15S-HETE with 12S-HETE being a minor side product (Fig. 9A+B). The corresponding enantiomers (15R-HETE, 12R-HETE) were virtually absent. However, for the Ile418Ala mutant an inverse product pattern was observed. For this enzyme variant 12S-HETE was the major AA oxygenation product and 15S-HETE was formed in smaller quantities (Fig. 9C + D). Here again, the corresponding enantiomers (15R-HETE, 12R-HETE) were virtually absent. When AA-Car was used as substrate the product patterns were very similar to those of free AA oxygenation. The dominant product of AA-Car oxygenation by wild-type rabbit ALOX15 was 15S-HETE but small amounts of 12S-HETE were also detected. As for free AA oxygenation the corresponding enantiomers (15R-HETE, 12R-HETE) were only detected in trace amounts (Fig. 9E + F). The Ile418Ala mutant of rabbit ALOX15 oxygenated AA-Car predominantly to the 12S-HETE derivative and 15S-HETE was detected in smaller amounts (Fig. 9G + H). Taken together, this data suggests that free AA and AA-Car are similarly aligned at the active site of wildtype rabbit ALOX15. In both cases the bisallylic methylene C_13_ of the AA moiety should be in close proximity to the non-heme iron so that hydrogen abstraction from C13 and thus, 15-lipoxygenation was strongly preferred. For the Ile418Ala mutant a different alignment of the two substrates was concluded. Here the bisallylic methylene C_10_ of the AA moiety should be in close proximity to the non-heme iron and thus 12-lipoxygenation was dominant.

**Fig. 10:**
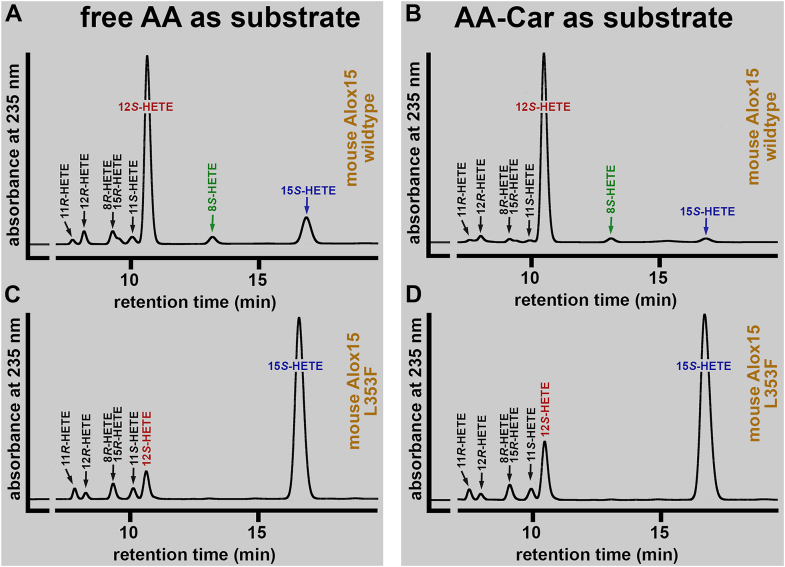
Product patterns formed from free AA and AA-Car by wildtype mouse Alox15 and its Leu353Phe mutant. Wild-type Mouse Alox15 and its Leu353Phe mutant were expressed in E. coli as recombinant hexa-His-tag fusion proteins and aliquots of the bacterial lysate supernatants were used as enzyme source for in vitro activity assays. The reaction products formed from free AA and AA-Car were prepared by RP-HPLC and further analyzed by combined NP/CP-HPLC as described in the Materials and Methods section. A: Wild-type mouse Alox15 with free AA as substrate. B: Wild-type mouse Alox15 with AA-Car as substrate. C: Leu353Phe mutant of mouse Alox15 with free AA as substrate. D: Leu353Phe mutant of mouse Alox15 with AA-Car as substrate.

Mouse Alox15 converts free AA predominantly to 12*S*-HETE (5), but Leu353Phe exchange humanized the reaction specificity of this enzyme with free AA (65). If AA-Car is aligned at the active site of mouse Alox15 in a similar way as free AA one would expect that the reaction specificity of AA-Car oxygenation is also altered, favoring 15-lipoxygenation. To test this hypothesis, we expressed wild-type mouse Alox15 and its Leu353Phe mutant as recombinant His-tag fusion protein, carried out in vitro activity assays and analyzed the major oxygenation products by NP/CP-HPLC. As expected, wild-type Alox15 oxygenated free AA mainly to 12S-HETE (Fig. 10A) and a similar product pattern was observed when AA-Car was used as substrate (Fig. 10B). For the Leu353Phe mutant 15S-HETE was identified as major AA oxygenation product (Fig. 10C) and this was also the case when AA-Car was used as substrate (Fig. 10D). Thus, Leu353Phe exchange altered the reaction specificities of wild-type mouse Alox15 with free AA and AA-Car in a similar way and this data suggests a similar alignment of the two substrates at the active site of the enzyme.

### In silico substrate docking studies and molecular dynamics simulations

**Fig. 11:**
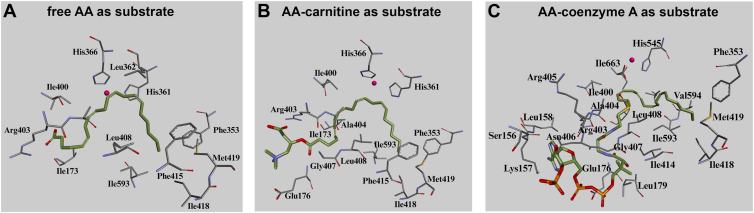
Docking poses of free AA, AA-Car and AA-CoA inside the substrate binding pocket of rabbit ALOX15. In silico docking studies were carried out as described in Materials and Methods. The alignments of the three substrates (green), the relevant amino acid side chains (stick model) and the catalytic non-heme iron (red sphere) are indicated: (A) free AA, (B) AA-Car, and (C) AA-CoA.

Our in vitro activity assays with rabbit ALOX15 (Fig. 1) indicated that the pure native enzyme is capable of oxygenating not only free AA but also AA-Car and AA-CoA to specific oxygenation products. For more detailed information on enzyme-substrate interaction, in particular on the substrate alignment at the active site of the enzyme, we next performed in silico docking studies to explore how well the three substrates (free AA, AA-Car, and AA-CoA) fit into the substrate binding pocket. For this purpose, we employed the X-ray coordinates of rabbit ALOX15 crystals. To determine the most plausible alignment of free AA inside the substrate binding pocket of the catalytic productive enzyme-substrate complex three initial assumptions were made: (i) The bisallylic carbon atom 13 (C_13_), which serves as primary target for initial hydrogen abstraction, was localized in close proximity to the non-heme iron. (ii) The negatively charged carboxylate group of free AA was placed in hydrogen bond distance of the negatively charged side chain of Arg403, which is consistent with previous mutagenesis studies (66). (iii) The methyl tail of the substrate fatty acid was placed in close proximity to the Triad determinants Ile418/Met419, Phe353, and Ile593 (47, 67), since mutagenesis of these residues altered the reaction specificity with both free AA and AA-Car (Fig. 11). Under these conditions free AA adopted a characteristic U-shaped conformation within the hydrophobic substrate binding pocket (Fig. 11A). This conformation was primarily stabilized by hydrophobic interactions of the fatty acid chain with the following amino acids: Ile173, Phe353, His361, Leu362, His366, Ile400, Ala404, Leu408, Phe415, Ile418, and Met419. The carboxylate moiety was in hydrogen bond distance to the flexible side chain of Arg403. Among these amino acids His361 and His366 serve as proteinogenic ligands for the catalytic non-heme iron (68, 69). Phe353 (70), Ile418, Met419 (71), and Ile593 (67) have previously been identified as Triad determinants, and side-directed mutagenesis of these amino acids in all mammalian ALOX15 orthologs tested so far altered the reaction specificity of the enzymes (47).

AA-Car also fits into the substrate binding pocket and no major steric constraints were observed. The substrate also adopts a U-shaped conformation inside the substrate binding pocket (Fig. 11B). The charged trimethylamine group formed an electrostatic interaction with Glu176, while the carboxylate moiety was positioned in close proximity to Arg403. The methyl end of the ligand was localized in proximity to the triad determinants (Ile418, Met419, and Ile593) together with the neighboring Phe415. The extended hydrophobic tail of AA-Car established multiple hydrophobic contacts with Ile173, His361 and His366 (proteinogenic iron ligands) Ile400, Ala404, Gly407, and Leu408 contributing to the overall stability of the binding mode.

AA-CoA is a much bulkier substrate than free AA and AA-Car. Nevertheless, according to the data of our comparative docking studies it fits into the substrate binding pocket of the enzyme, but its alignment is completely different from that of free AA and AA-Car. The position of the aliphatic fatty acid chain is stabilized by hydrophobic interactions with the side chains of Ile173, Phe353, Ile400, Ile414, Ile418, Met419, Ile593, Val594 and Ile663. Amino acids given in bold did also contribute to aligning free AA at the active site of the enzyme (Fig. 11A). The more hydrophilic CoA moiety of AA-CoA occupies a more solvent-exposed region of the substrate binding pocket and interacts with Leu158, Lys157, and Arg405. Notably, Lys157 formed a salt bridge, while Leu158 and Arg405 were involved in hydrogen bond interactions. However, despite these favorable interactions, the bulky nature of the ligand resulted in multiple steric clashes with Glu176, Leu179, Arg403, Asn406, Gly407, Leu408, and Leu597, suggesting higher entropic contributions than the other two substrates.

To test the stability of the enzyme-substrate complexes, we preformed MD simulations and found that the U-shaped conformation of AA was maintained within the active site of ALOX15 throughout the simulation period (supplemental movie M1). Arg403 emerged as a key residue, consistently forming a hydrogen bond throughout the trajectory. Interestingly, the carboxylate moiety of free AA formed water-mediated contacts with Ile173 and Glu176. The hydrophobic methyl tail of free AA remained in close proximity to several key residues including the Triad determinants (Phe353, Ile418+Met419, and Ile593) and Ile414. The U-shaped conformation of AA-Car at the active site of rabbit ALOX15 was also stable throughout the simulation period (supplemental movie M2). The esterified carboxylate group of the ligand formed multiple interactions with the side chains of Asn406, Glu176, Ser178, and Arg403. A dynamic flipping of the carboxylate group was observed during the simulation, which ultimately stabilized via prominent hydrogen bonding with the side chain of Arg403, further reinforcing the ligand’s binding pose. The C_13_ atom consistently remained in close proximity to the non-heme iron atom suggesting a catalytically favorable substrate alignment. The methyl end of the ligand remained in close contact with the catalytic triad (Phe353, Ile418+Met419, and Ile593) throughout the entire trajectory.

In contrast, the ALOX15-AA-CoA complex (Fig. 11C) was less stable and exhibited a higher degree of motional flexibility (supplemental movie M3). Here again, the fatty acid moiety interacted via hydrophobic interactions with the side chains of His366 (iron ligand), Leu362, Phe353 (Triad determinant), Ile414, Ile418 (Triad determinant), Met419 (Triad determinant), and Ile593 (Triad determinant). Overall, the aliphatic hydrocarbon chain of the substrate maintained a U-shaped conformation near the non-heme iron, facilitating hydrogen abstraction from carbon 13. The bulky CoA moiety was positioned close to Ser156, Lys157, Val402, Arg403, Arg405, Asn406, Ser410, and Asp411. During the simulation, the nitrogenous base of the adenosine group flipped, and a stable hydrogen bond was formed between the positively charged side chain of Lys157 and the phosphate group of the substrate. The amide group of the 4-phosphopantothenate formed a hydrogen bond with the side chain of Asn406. Toward the end of the simulation period the amine (-NH_2_) group of the nitrogenous base approached the main chain of Val402 resulting in the formation of a stable hydrogen bond. In addition, Ser156 established water-mediated contacts with the phosphate group.

In summary, our docking studies together with the MD simulations indicate that free AA and AA-Car are similarly aligned at the active site of the enzyme and this data explains the relatively high oxygenase activities of the enzyme with these two substrates. In contrast, we observed a number of steric constraints and a higher degree of motional flexibility for the binding of AA-CoA, which may contribute to the low AA-CoA oxygenase activity of the enzyme.

## Discussion

### Degree of novelty and advancement of science

According to the canonical concept of the AA cascade ALOX isoforms have been implicated in the formation of bioactive lipid mediators (10, 11). ALOX catalyzed oxygenation of free polyenoic fatty acids leads to the formation of eicosanoids and related compounds such as leukotrienes (72), eoxins (73), hepoxilins (16), lipoxins (74), resolvins (21), and others. However, some ALOX-isoforms are capable of oxygenating more complex substrates such as phospholipids (26) and cholesterol esters (75) even if these compounds are incorporated in complex lipid-protein assemblies, such as biomembranes (30) and lipoproteins (76). More recently, it has been shown that ALOX isoforms are capable of oxygenating endocannabinoids such as anandamide and 2-arachidonoyl glycerol with higher efficiency than free AA (57). This result was somewhat surprising since previous experiments suggested that PUFA methyl esters are less suitable substrates for these enzymes (56).

In mammalian cells, free PUFA concentrations are rather low since free fatty acids liberated from the cellular ester lipids are rapidly re-esterified to acyl-CoA esters via the catalytic activity of evolutionary highly conserved acyl-CoA synthetases. Stearoyl-CoA and oleoyl-CoA esters have recently been identified as allosteric ALOX inhibitors (46) and this data suggested an interaction of these lipids with the ALOX proteins. However, it has not been explored whether these lipids are capable of entering the active site of the enzymes since the bulky CoA moiety might cause steric clashes. Moreover, it appeared unlikely that the charged CoA moiety is capable of penetrating into the hydrophobic environment of the substrate binding pocket. In the present study, we extended our knowledge on the substrate specificity of mammalian ALOX-isoforms in two different aspects.

i) We showed that mouse and human ALOX15 orthologs, mouse and human ALOX15B orthologs and human ALOX12 are capable of oxygenating AA-carnitine and AA-CoA without the preceding activity of an ester lipid hydrolyzing enzyme. Side-by-side comparative experiments indicated that rabbit ALOX15, human ALOX15, mouse Alox15 and human ALOX15B oxygenate AA-Car with similar or higher rates than free AA. In contrast, for mouse Alox15B and human ALOX12 the oxygenation rates for AA-Car were somewhat lower than those of free AA oxygenation. Nevertheless, these enzymes do also exhibit an AA-Car oxygenase activity. Inter-enzyme comparison (Fig. 4A) indicated that when normalized to identical AA oxygenase activities rabbit ALOX15 exhibited the highest AA-Car oxygenase activity followed by human ALOX15, mouse ALOX15 and human ALOX15B. For the AA-CoA ester the situation was somewhat different. Although all enzymes tested exhibited an AA-CoA ester oxygenase activity much higher (5-10-fold) enzymes concentrations were needed to prepare sufficient amounts of oxygenation products. For this substrate direct inter-enzyme comparison (Fig. 4B + C) indicated that when normalized to a similar AA oxygenase activity human ALOX15, mouse Alox15 and mouse Alox15b were similarly effective followed by human ALOX15B and human ALOX12.
ii) Since the reaction of mammalian ALOX-isoforms with acyl-carnitines and acyl-CoA esters has not been tested before, it was completely unclear which products are formed during this reaction. We found that rabbit ALOX15, human ALOX15, human ALOX15B, and mouse Alox15 oxygenated AA-Car to similar product patterns as free AA (Fig. 6). In contrast, mouse Alox15b and human ALOX12 showed interesting differences. In fact, mouse Alox15b, which oxygenates free AA almost exclusively to 8S-HETE (63), converted AA-Car predominantly to the 15S-HETE derivative. Human ALOX12, which oxygenates free AA with singular reaction specificity to 12S-HETE (62, 77), exhibits a dual reaction specificity with AA-Car forming similar amounts of 12S- HETE and 15S-HETE. With AA-CoA as substrate similar isoform-specific product patterns were observed. Here, mouse Alox15, human ALOX15B and human ALOX12 form similar patterns of oxygenation products when free AA was compared with AA-CoA (Fig. 7). In contrast, substrate-specific differences were observed for the product patterns formed by human ALOX15 and mouse Alox15b.

### Possible biological relevance of ALOX-catalyzed oxygenation of arachidonoyl-coenzyme A and arachidonoyl-carnitine

When fatty acids are liberated from the membrane phospholipids, their half-life as free fatty acids is rather short. They are rapidly re-esterified to acyl-CoA esters via the catalytic activity of acyl-CoA synthetases using ATP as energy source (78). Alternatively (10), they are oxygenated to eicosanoids and related compounds via the catalytic activity of prostaglandin synthase, lipoxygenase or cytochrome P450 isoforms (Fig. 1). Eicosanoids and related compounds are liberated from the cells and function as extracellular lipid mediators modifying the functional phenotype of target cells. Acyl CoA esters are not liberated from the cells but are further metabolized intracellularly via alternative pathways: i) They rapidly react with membrane-bound lysophosphatides (45) forming new membrane phospholipids (Lands cycle). ii) In adipocytes they constitute substrates for triacylglycerol biosynthesis (79). iii) They are trans-esterified to acyl carnitines, which are subsequently imported into the mitochondria fueling ß-oxidation (80). iv) They undergo desaturation (81) and/or elongation (82) altering the length of the hydrocarbon chain and their degree of unsaturation. In most resting mammalian cells acyl-CoA esters and acyl-carnitines occur much more abundantly than free fatty acids and thus, HETE-containing carnitines and HETE-containing CoA esters should be present in ALOX-expressing cells. Unfortunately, for the time being it has never been tested whether these products may occur in vivo, whether they are of biological relevance and whether there are differences in the biological roles of the different positional isomers (comparison of the bioactivity of 15-HETE-carnitin vs. 12-HETE-carnitine). Although AA-CoA is a less effective ALOX substrate, the same questions are relevant for its oxygenation product.

The major biological relevance of acyl carnitines is mitochondrial import of fatty acids. When fatty acids are employed as energy source they need to be imported into the mitochondria where they are degraded via ß-oxidation to acetyl-CoA units (83), which are subsequently oxidized in the Krebs cycle. Since mitochondrial inner and outer membranes are not permeable for free fatty acids the acyl chains must be converted to acyl carnitines, which are subsequently transported into the mitochondrial matrix via the carnitine-acyl carnitine exchanger (84). Although these membrane transporters have been well-characterized with respect to their substrate preference, it has never been tested whether they also accept HETE-carnitines as substrates. If so, the HETE-carnitines can be imported into mitochondria and used as substrates for ß-oxidation (85). If not, HETE-carnitines may be catabolized intracellularly via alternative pathways (peroxisomal ß-oxidation) or be released from the cells to function as lipid signaling molecules.

### Limitations of the study and further research directions

To test whether AA-Car and AA-CoA constitute ALOX substrates, we incubated selected ALOX-isoforms with the two substrates in vitro and analyzed the hydrolyzed lipid extracts for the presence of HETE-isomers. In additional experiments we also analyzed the non-hydrolyzed lipid extracts of our in vitro activity assays by RP-HPLC, but we never detected symmetric peaks of conjugated dienes. This analytical failure might be related to the fact that non-hydrolyzed acyl carnitines and acyl CoA esters are charged molecules and thus, may differently interact with the chromatographic matrix than free HETE-isomers. In other words, reliable analysis of HETE-containing carnitines and CoA esters requires a specialized analytical protocol and major methodological development, which exceed the frame of the present study.

When we compared the substrate efficiencies of free AA, AA-car, and AA-CoA for the different ALOX-isoforms, we found that for human and mouse ALOX15 and ALOX15B orthologs free AA and AA-car were oxygenated with similar efficiencies (Fig. 3A, C, E, G). In contrast, for human ALOX12 free AA was a much better substrate (Fig. 3I). However, it should be stressed that these substrate rankings were not based on detailed kinetic studies since the use of crude enzyme preparations did not allow precise quantification of kinetic constants, such as k_cat_. Nevertheless, our data suggest that under strictly comparable conditions AA-car appears to be a good substrate for mammalian ALOX15 and ALOX15B orthologs but not for ALOX12.

The data presented in this study indicate that in reconstituted molecular assay systems AA-Car and AA-CoA are suitable substrates for mammalian ALOX isoforms. However, whether HETE-Car and HETE-CoA derivatives are also formed in cells, in organ preparations or in whole animals has not been tested. The major reason for this limitation was that our attempts to reliably quantify HETE-carnitines and HETE-CoA esters were not successful. We applied our methodology (HPLC analysis of the hydrolyzed lipid extracts) to ALOX15 expressing L1236 lymphoma cells (86) and observed the presence of 15-HETE. Unfortunately, since our alkaline hydrolysis method was unselective this 15-HETE might originate from any 15-HETE containing ester lipid. Thus, our analytical system was just not suitable to answer the question whether HETE-Car and HETE-CoA esters are formed in ALOX expressing in vivo systems.

The biological relevance of the HETE-Car and HETE-CoA esters, which may be formed via direct oxygenation of AA-Car and AA-CoA by different ALOX-isoforms, has not been explored in the present study. However, based on our findings such questions can be investigated in follow-up experiments. We showed that native and recombinant ALOX-isoforms are capable of oxygenating AA-car, AA-CoA, and probably the corresponding esters of other polyunsaturated fatty acids to highly specific oxygenation products. In other words, these enzymes can be used to prepare mg amounts of specifically oxygenated PUFA-containing carnitines and PUFA-containing CoA esters, which can subsequently be tested for their biological functions in different cellular assay systems. Moreover, these complex oxylipins can also be used as reference compounds to explore whether these products are formed in cellular in vitro systems or in whole animal disease models. We are currently trying to identify HETE-containing carnitines and HETE-containing CoA esters in ALOX15 expressing L1236 human lymphoma cells (87) using ALOX15-deficient lymphoma cells as negative controls. Similar experiments will be carried out with wild-type mouse peritoneal macrophages, with corresponding cells of Alox15^−/−^ mice (5) and humanized Alox15 knock-in mice (65).

### Data availability

The experimental raw data obtained in this study can be obtained upon request from HK and PA.

## Supplemental data

This article contains supplemental data.

## Conflict of interest

The authors declare that they do not have any conflicts of interest with the content of this article.

## References

[1] Haeggstrom J.Z., Funk C.D. (2011). Lipoxygenase and leukotriene pathways: biochemistry, biology, and roles in disease. Chem. Rev..

[2] Ivanov I., Heydeck D., Hofheinz K., Roffeis J., O'Donnell V.B., Kuhn H. (2010). Molecular enzymology of lipoxygenases. Arch. Biochem. Biophys..

[3] Mashima R., Okuyama T. (2015). The role of lipoxygenases in pathophysiology; new insights and future perspectives. Redox Biol..

[4] Funk C.D., Chen X.S., Johnson E.N., Zhao L. (2002). Lipoxygenase genes and their targeted disruption. Prostaglandins Other Lipid Mediat..

[5] Sun D., Funk C.D. (1996). Disruption of 12/15-lipoxygenase expression in peritoneal macrophages. Enhanced utilization of the 5-lipoxygenase pathway and diminished oxidation of low density lipoprotein. J. Biol. Chem..

[6] Johnson E.N., Brass L.F., Funk C.D. (1998). Increased platelet sensitivity to ADP in mice lacking platelet-type 12-lipoxygenase. Proc. Natl. Acad. Sci. U. S. A..

[7] Zhao L., Moos M.P., Grabner R., Pedrono F., Fan J., Kaiser B. (2004). The 5-lipoxygenase pathway promotes pathogenesis of hyperlipidemia-dependent aortic aneurysm. Nat. Med..

[8] Epp N., Furstenberger G., Muller K., de Juanes S., Leitges M., Hausser I. (2007). 12R-lipoxygenase deficiency disrupts epidermal barrier function. J. Cell Biol..

[9] Krieg P., Rosenberger S., de Juanes S., Latzko S., Hou J., Dick A. (2013). Aloxe3 knockout mice reveal a function of epidermal lipoxygenase-3 as hepoxilin synthase and its pivotal role in barrier formation. J. Invest. Dermatol..

[10] Hanna V.S., Hafez E.A.A. (2018). Synopsis of arachidonic acid metabolism: a review. J. Adv. Res..

[11] Bosetti F. (2007). Arachidonic acid metabolism in brain physiology and pathology: lessons from genetically altered mouse models. J. Neurochem..

[12] Aloulou A., Rahier R., Arhab Y., Noiriel A., Abousalham A. (2018). Phospholipases: an overview. Methods Mol. Biol..

[13] Liu M., Yokomizo T. (2015). The role of leukotrienes in allergic diseases. Allergol. Int..

[14] Drazen J.M. (2002). Anti-leukotrienes as novel anti-inflammatory treatments in asthma. Adv. Exp. Med. Biol..

[15] Amlani S., Nadarajah T., McIvor R.A. (2011). Montelukast for the treatment of asthma in the adult population. Expert Opin. Pharmacother..

[16] Pace-Asciak C.R. (2015). Pathophysiology of the hepoxilins. Biochim. Biophys. Acta.

[17] Brash A.R., Yu Z., Boeglin W.E., Schneider C. (2007). The hepoxilin connection in the epidermis. FEBS J..

[18] Pace-Asciak C.R. (2009). The hepoxilins and some analogues: a review of their biology. Br. J. Pharmacol..

[19] Sachs-Olsen C., Sanak M., Lang A.M., Gielicz A., Mowinckel P., Lodrup Carlsen K.C. (2010). Eoxins: a new inflammatory pathway in childhood asthma. J. Allergy Clin. Immunol..

[20] Hu S., Mao-Ying Q.L., Wang J., Wang Z.F., Mi W.L., Wang X.W. (2012). Lipoxins and aspirin-triggered lipoxin alleviate bone cancer pain in association with suppressing expression of spinal proinflammatory cytokines. J. Neuroinflammation.

[21] Serhan C.N., Petasis N.A. (2011). Resolvins and protectins in inflammation resolution. Chem. Rev..

[22] Dalli J., Colas R.A., Serhan C.N. (2013). Novel n-3 immunoresolvents: structures and actions. Sci. Rep..

[23] Serhan C.N., Dalli J., Colas R.A., Winkler J.W., Chiang N. (2015). Protectins and maresins: new pro-resolving families of mediators in acute inflammation and resolution bioactive metabolome. Biochim. Biophys. Acta.

[24] Kahnt A.S., Hafner A.K., Steinhilber D. (2024). The role of human 5-Lipoxygenase (5-LO) in carcinogenesis - a question of canonical and non-canonical functions. Oncogene.

[25] Brash A.R., Ingram C.D., Harris T.M. (1987). Analysis of a specific oxygenation reaction of soybean lipoxygenase-1 with fatty acids esterified in phospholipids. Biochemistry.

[26] Schewe T., Halangk W., Hiebsch C., Rapoport S.M. (1975). A lipoxygenase in rabbit reticulocytes which attacks phospholipids and intact mitochondria. FEBS Lett..

[27] Rapoport S.M., Schewe T., Wiesner R., Halangk W., Ludwig P., Janicke-Hohne M. (1979). The lipoxygenase of reticulocytes. Purification, characterization and biological dynamics of the lipoxygenase; its identity with the respiratory inhibitors of the reticulocyte. Eur. J. Biochem..

[28] Kühn H., Barnett J., Grunberger D., Baecker P., Chow J., Nguyen B. (1993). Overexpression, purification and characterization of human recombinant 15-lipoxygenase. Biochim. Biophys. Acta.

[29] Bender G., Schexnaydre E.E., Murphy R.C., Uhlson C., Newcomer M.E. (2016). Membrane-dependent activities of human 15-LOX-2 and its murine counterpart: implications for murine models of atherosclerosis. J. Biol. Chem..

[30] Kuhn H., Belkner J., Wiesner R., Brash A.R. (1990). Oxygenation of biological membranes by the pure reticulocyte lipoxygenase. J. Biol. Chem..

[31] Belkner J., Wiesner R., Rathman J., Barnett J., Sigal E., Kuhn H. (1993). Oxygenation of lipoproteins by mammalian lipoxygenases. Eur. J. Biochem..

[32] Rapoport S.M., Schewe T. (1986). The maturational breakdown of mitochondria in reticulocytes. Biochim. Biophys. Acta.

[33] Rademacher M., Kuhn H., Borchert A. (2020). Systemic deficiency of mouse arachidonate 15-lipoxygenase induces defective erythropoiesis and transgenic expression of the human enzyme rescues this phenotype. FASEB J..

[34] van Leyen K., Duvoisin R.M., Engelhardt H., Wiedmann M. (1998). A function for lipoxygenase in programmed organelle degradation. Nature.

[35] Pernet E., Sun S., Sarden N., Gona S., Nguyen A., Khan N. (2023). Neonatal imprinting of alveolar macrophages via neutrophil-derived 12-HETE. Nature.

[36] Krieg P., Furstenberger G. (2014). The role of lipoxygenases in epidermis. Biochim. Biophys. Acta.

[37] Eckl K.M., de Juanes S., Kurtenbach J., Natebus M., Lugassy J., Oji V. (2009). Molecular analysis of 250 patients with autosomal recessive congenital ichthyosis: evidence for mutation hotspots in ALOXE3 and allelic heterogeneity in ALOX12B. J. Invest. Dermatol..

[38] Akiyama M. (2006). Harlequin ichthyosis and other autosomal recessive congenital ichthyoses: the underlying genetic defects and pathomechanisms. J. Dermatol. Sci..

[39] Cao Y., Pearman A.T., Zimmerman G.A., McIntyre T.M., Prescott S.M. (2000). Intracellular unesterified arachidonic acid signals apoptosis. Proc. Natl. Acad. Sci. U. S. A..

[40] Serini S., Trombino S., Oliva F., Piccioni E., Monego G., Resci F. (2008). Docosahexaenoic acid induces apoptosis in lung cancer cells by increasing MKP-1 and down-regulating p-ERK1/2 and p-p38 expression. Apoptosis.

[41] Ellis J.M., Frahm J.L., Li L.O., Coleman R.A. (2010). Acyl-coenzyme A synthetases in metabolic control. Curr. Opin. Lipidol..

[42] Kuwata H., Hara S. (2019). Role of acyl-CoA synthetase ACSL4 in arachidonic acid metabolism. Prostaglandins Other Lipid Mediat..

[43] Watkins P.A., Maiguel D., Jia Z., Pevsner J. (2007). Evidence for 26 distinct acyl-coenzyme A synthetase genes in the human genome. J. Lipid Res..

[44] Cao Y., Traer E., Zimmerman G.A., McIntyre T.M., Prescott S.M. (1998). Cloning, expression, and chromosomal localization of human long-chain fatty acid-CoA ligase 4 (FACL4). Genomics.

[45] O'Donnell V.B. (2022). New appreciation for an old pathway: the Lands Cycle moves into new arenas in health and disease. Biochem. Soc. Trans..

[46] Tran M., Yang K., Glukhova A., Holinstat M., Holman T. (2023). Inhibitory investigations of acyl-CoA derivatives against human lipoxygenase isozymes. Int. J. Mol. Sci..

[47] Heydeck D., Reisch F., Schafer M., Kakularam K.R., Roigas S.A., Stehling S. (2022). The reaction specificity of mammalian ALOX15 orthologs is changed during late primate evolution and these alterations might offer evolutionary advantages for hominidae. Front. Cell Dev. Biol..

[48] Goloshchapova K., Stehling S., Heydeck D., Blum M., Kuhn H. (2019). Functional characterization of a novel arachidonic acid 12S-lipoxygenase in the halotolerant bacterium Myxococcus fulvus exhibiting complex social living patterns. Microbiologyopen.

[49] Bligh E.G., Dyer W.J. (1959). A rapid method of total lipid extraction and purification. Can J. Biochem. Physiol..

[50] Kuhn H., Humeniuk L., Kozlov N., Roigas S., Adel S., Heydeck D. (2018). The evolutionary hypothesis of reaction specificity of mammalian ALOX15 orthologs. Prog. Lipid Res..

[51] Choi J., Chon J.K., Kim S., Shin W. (2008). Conformational flexibility in mammalian 15S-lipoxygenase: reinterpretation of the crystallographic data. Proteins.

[52] Frisch M.J., Trucks G.W., Schlegel H.B., Scuseria G.E., Robb M.A., Cheeseman J.R. (2016).

[53] Jones G., Willett P., Glen R.C., Leach A.R., Taylor R. (1997). Development and validation of a genetic algorithm for flexible docking. J. Mol. Biol..

[54] Toledo L., Masgrau L., Maréchal J.D., Lluch J.M., González-Lafont A. (2010). Insights into the mechanism of binding of arachidonic acid to mammalian 15-lipoxygenases. J. Phys. Chem. B.

[55] Bowers K.J., Chow D.E., Xu H., Dror R.O., Eastwood M.P., Gregersen B.A. (2006). Scalable algorithms for molecular dynamics simulations on commodity clusters. SC '06: Proceedings of the 2006 ACM/IEEE Conference on Supercomputing.

[56] Schwarz K., Borngraber S., Anton M., Kuhn H. (1998). Probing the substrate alignment at the active site of 15-lipoxygenases by targeted substrate modification and site-directed mutagenesis. Evidence for an inverse substrate orientation. Biochemistry.

[57] Ivanov I., Kakularam K.R., Shmendel E.V., Rothe M., Aparoy P., Heydeck D. (2021). Oxygenation of endocannabinoids by mammalian lipoxygenase isoforms. Biochim. Biophys. Acta Mol. Cell Biol. Lipids.

[58] Bryant R.W., Bailey J.M., Schewe T., Rapoport S.M. (1982). Positional specificity of a reticulocyte lipoxygenase. Conversion of arachidonic acid to 15-S-hydroperoxy-eicosatetraenoic acid. J. Biol. Chem..

[59] Kuhn H., Wiesner R., Schewe T., Rapoport S.M. (1983). Reticulocyte lipoxygenase exhibits both n-6 and n-9 activities. FEBS Lett..

[60] Brash A.R., Boeglin W.E., Chang M.S. (1997). Discovery of a second 15S-lipoxygenase in humans. Proc. Natl. Acad. Sci. U. S. A..

[61] Furstenberger G., Hagedorn H., Jacobi T., Besemfelder E., Stephan M., Lehmann W.D. (1991). Characterization of an 8-lipoxygenase activity induced by the phorbol ester tumor promoter 12-O-tetradecanoylphorbol-13-acetate in mouse skin in vivo. J. Biol. Chem..

[62] Hamberg M., Samuelsson B. (1974). Prostaglandin endoperoxides. Novel transformations of arachidonic acid in human platelets. Proc. Natl. Acad. Sci. U. S. A..

[63] Jisaka M., Kim R.B., Boeglin W.E., Brash A.R. (2000). Identification of amino acid determinants of the positional specificity of mouse 8S-lipoxygenase and human 15S-lipoxygenase-2. J. Biol. Chem..

[64] Vogel R., Jansen C., Roffeis J., Reddanna P., Forsell P., Claesson H.E. (2010). Applicability of the triad concept for the positional specificity of mammalian lipoxygenases. J. Biol. Chem..

[65] Reisch F., Heydeck D., Schafer M., Rothe M., Yang J., Stehling S. (2023). Knock-in mice expressing a humanized arachidonic acid 15-lipoxygenase (Alox15) carry a partly dysfunctional erythropoietic system. Cell Mol. Biol. Lett..

[66] Gan Q.F., Browner M.F., Sloane D.L., Sigal E. (1996). Defining the arachidonic acid binding site of human 15-lipoxygenase. Molecular modeling and mutagenesis. J. Biol. Chem..

[67] Borngraber S., Browner M., Gillmor S., Gerth C., Anton M., Fletterick R. (1999). Shape and specificity in mammalian 15-lipoxygenase active site. The functional interplay of sequence determinants for the reaction specificity. J. Biol. Chem..

[68] Gillmor S.A., Villasenor A., Fletterick R., Sigal E., Browner M.F. (1997). The structure of mammalian 15-lipoxygenase reveals similarity to the lipases and the determinants of substrate specificity. Nat. Struct. Biol..

[69] Kuban R.J., Wiesner R., Rathman J., Veldink G., Nolting H., Solé V.A. (1998). The iron ligand sphere geometry of mammalian 15-lipoxygenases. Biochem. J..

[70] Borngraber S., Kuban R.J., Anton M., Kuhn H. (1996). Phenylalanine 353 is a primary determinant for the positional specificity of mammalian 15-lipoxygenases. J. Mol. Biol..

[71] Sloane D.L., Leung R., Craik C.S., Sigal E. (1991). A primary determinant for lipoxygenase positional specificity. Nature.

[72] Kanaoka Y., Boyce J.A. (2014). Cysteinyl leukotrienes and their receptors; emerging concepts. Allergy Asthma Immunol. Res..

[73] Feltenmark S., Gautam N., Brunnstrom A., Griffiths W., Backman L., Edenius C. (2008). Eoxins are proinflammatory arachidonic acid metabolites produced via the 15-lipoxygenase-1 pathway in human eosinophils and mast cells. Proc. Natl. Acad. Sci. U. S. A..

[74] Romano M. (2010). Lipoxin and aspirin-triggered lipoxins. ScientificWorldJournal.

[75] Belkner J., Wiesner R., Kuhn H., Lankin V.Z. (1991). The oxygenation of cholesterol esters by the reticulocyte lipoxygenase. FEBS Lett..

[76] Belkner J., Stender H., Kuhn H. (1998). The rabbit 15-lipoxygenase preferentially oxygenates LDL cholesterol esters, and this reaction does not require vitamin E. J. Biol. Chem..

[77] Nugteren D.H. (1975). Arachidonate lipoxygenase in blood platelets. Biochim. Biophys. Acta.

[78] Soupene E., Kuypers F.A. (2008). Mammalian long-chain Acyl-CoA synthetases. Exp. Biol. Med..

[79] Chu T., Yang M.S. (2023). A review of structural features, biological functions and biotransformation studies in adipose tissues and an assessment of progress and implications. Endocr. Metab. Immune Disord. Drug Targets.

[80] Nakamura M.T., Yudell B.E., Loor J.J. (2014). Regulation of energy metabolism by long-chain fatty acids. Prog. Lipid Res..

[81] Lee J.M., Lee H., Kang S., Park W.J. (2016). Fatty acid desaturases, polyunsaturated fatty acid regulation, and biotechnological advances. Nutrients.

[82] Wang X., Yu H., Gao R., Liu M., Xie W. (2023). A comprehensive review of the family of very-long-chain fatty acid elongases: structure, function, and implications in physiology and pathology. Eur. J. Med. Res..

[83] Eaton S., Bartlett K., Pourfarzam M. (1996). Mammalian mitochondrial beta-oxidation. Biochem. J..

[84] Bonnefont J.P., Djouadi F., Prip-Buus C., Gobin S., Munnich A., Bastin J. (2004). Carnitine palmitoyltransferases 1 and 2: biochemical, molecular and medical aspects. Mol. Aspects Med..

[85] Feussner I., Balkenhohl T.J., Porzel A., Kuhn H., Wasternack C. (1997). Structural elucidation of oxygenated storage lipids in cucumber cotyledons. Implication of lipid body lipoxygenase in lipid mobilization during germination. J. Biol. Chem..

[86] Liu C., Schain F., Han H., Xu D., Andersson-Sand H., Forsell P. (2012). Epigenetic and transcriptional control of the 15-lipoxygenase-1 gene in a Hodgkin lymphoma cell line. Exp. Cell Res..

[87] Han H., Xu D., Liu C., Claesson H.E., Bjorkholm M., Sjoberg J. (2014). Interleukin-4-mediated 15-lipoxygenase-1 trans-activation requires UTX recruitment and H3K27me3 demethylation at the promoter in A549 cells. PLoS One.

