# Supplementary material for: Arachidonoyl-carnitine and arachidonoyl-coenzyme A are suitable substrates for mammalian ALOX isoforms
Source: J Lipid Res. 2025 Jul 17;66(8):100861. doi: 10.1016/j.jlr.2025.100861 (PMC12396440; doi:10.1016/j.jlr.2025.100861)
Supplement: Supplemental data [file mmc1.docx]

**Supplemental information for the paper**

**Arachidonoyl-carnitine and arachidonoyl-coenzyme A are suitable substrates for mammalian ALOX isoforms.**

Xin Chen^1^, Sahanawaz Parvez^2^, Hannah F. Wiegand^1^, ^3^Liuhui Wu, Sabine Stehling^1^, Astrid Borchert^1^, Junlin Yang^4^, Polamarasetty Aparoy^2^ and Hartmut Kuhn^1*^

^1^Charité – Universitätsmedizin Berlin, corporate member of Freie Universität Berlin and Humboldt Universität zu Berlin, Department of Biochemistry, Charitéplatz 1, D-10117 Berlin, Germany.

^2^Molecular Modeling and Protein Engineering Lab, Biology Division, Department of Humanities and Sciences, Indian Institute of Petroleum and Energy, Visakhapatnam, Andhra Pradesh, 530003, India

^3^Spine Center, Xinhua Hospital Affiliated to Shanghai Jiao Tong University School of Medicine, Shanghai, China

^4^Department of Radiation Oncology, Cancer Center, The First Affiliated Hospital, Sun Yat-Sen University, Guangzhou, China

**Running title:** Acyl-carnitines and coenzyme A-esters as lipoxygenase substrates

***Correspondence to:** hartmut.kuehn@charite.de

**Funding:** The China Scholarship Council (CSC) supported this research by providing the following research grant: 202106380080

**Figure S1: Heat inactivation of rabbit ALOX15 prevented oxygenation of AA-carnitine and AA-CoA.** The solution of pure rabbit ALOX15 was incubated for 5 min at 95° C and aliquots of the native and the denatured enzymes were used for in vitro activity assays (see Materials and methods for details). Conjugated dienes were detected in the native enzyme incubations (panels A+B) but not in the samples of the heated enzyme preparations (panels C+D).

**
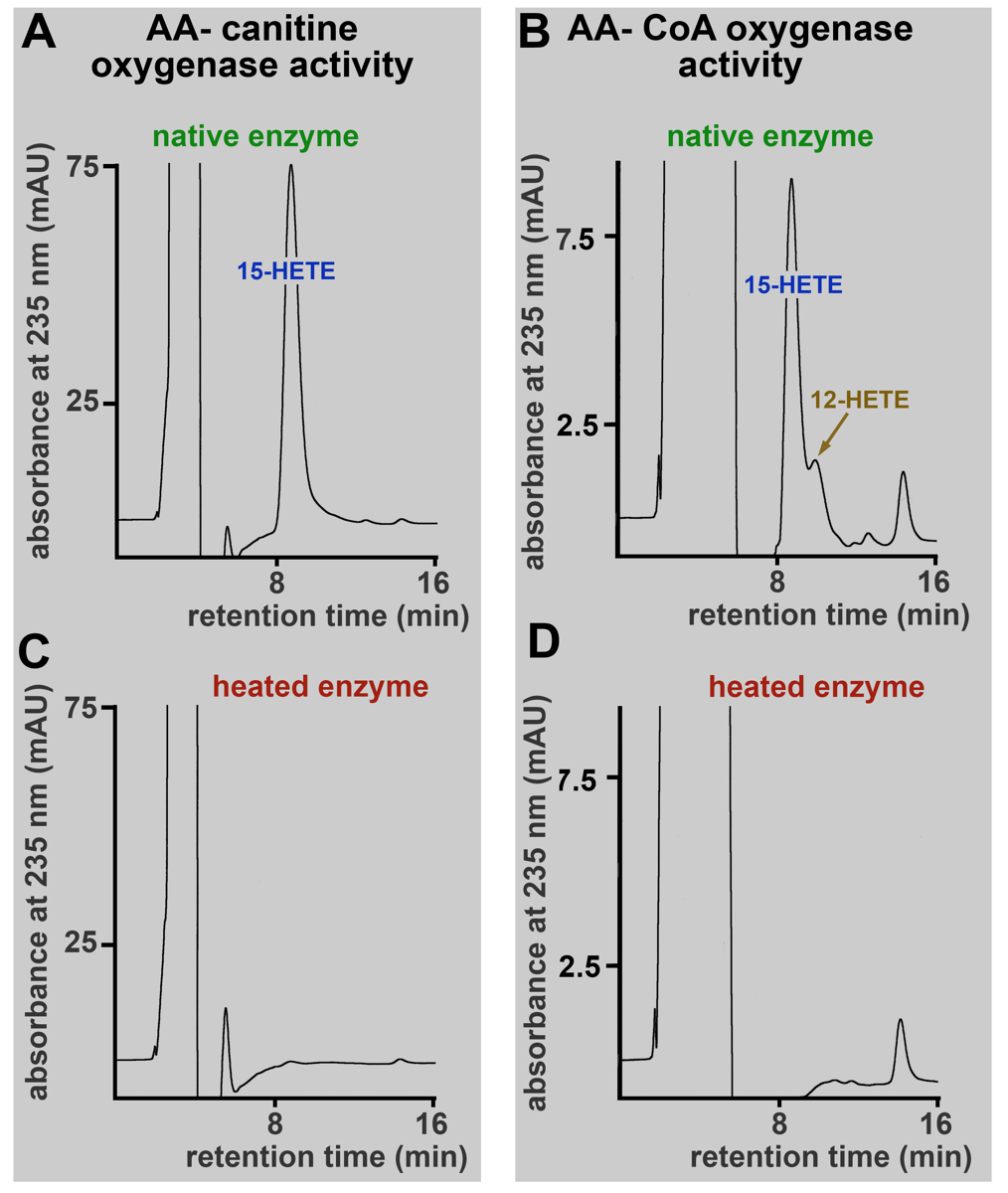
**

**Figure S2: Disappearance of substrate during in vitro activity assays.** In vitro activity assays were carried out with free AA, AA-Car and AA-CoA at three different enzyme concentrations [no enzyme, low enzyme (3 µl), high enzyme (30 µl)]. After sample work-up (see Materials and methods) the hydrolyzed lipid extracts were analyzed by RP-HPLC and the chromatograms were followed at 210 nm. The AA peaks were quantified. For each substrate the y-axis was scaled to the height of the “no enzyme” AA peak (100 % substrate, panels A, D and G). At low enzyme concentrations (panels B, E, and H) more than 50 % of the substrates were still present in the incubation sample. At high enzyme concentrations (panels C, F and I) more than 90 % of substrates were consumed.

**
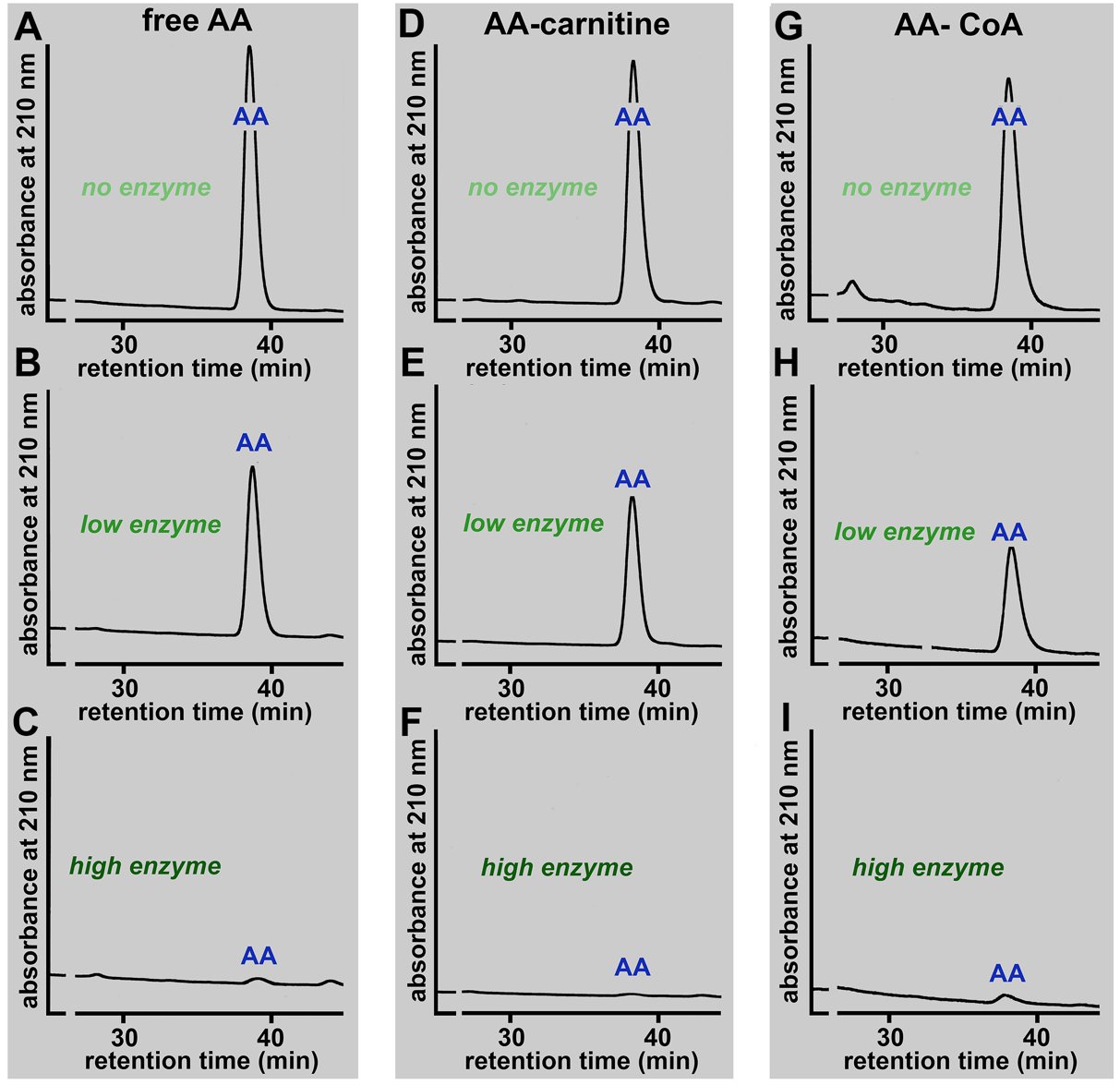
**

**Supplemental movie M1: MD simulation of AA binding at the active site of rabbit ALOX15.** In silico docking studies on the binding of free AA at the active site of rabbit ALOX15 and MD-simulations were carried out as described in the Materials and Methods section. A 100 ns simulation period is shown.

**
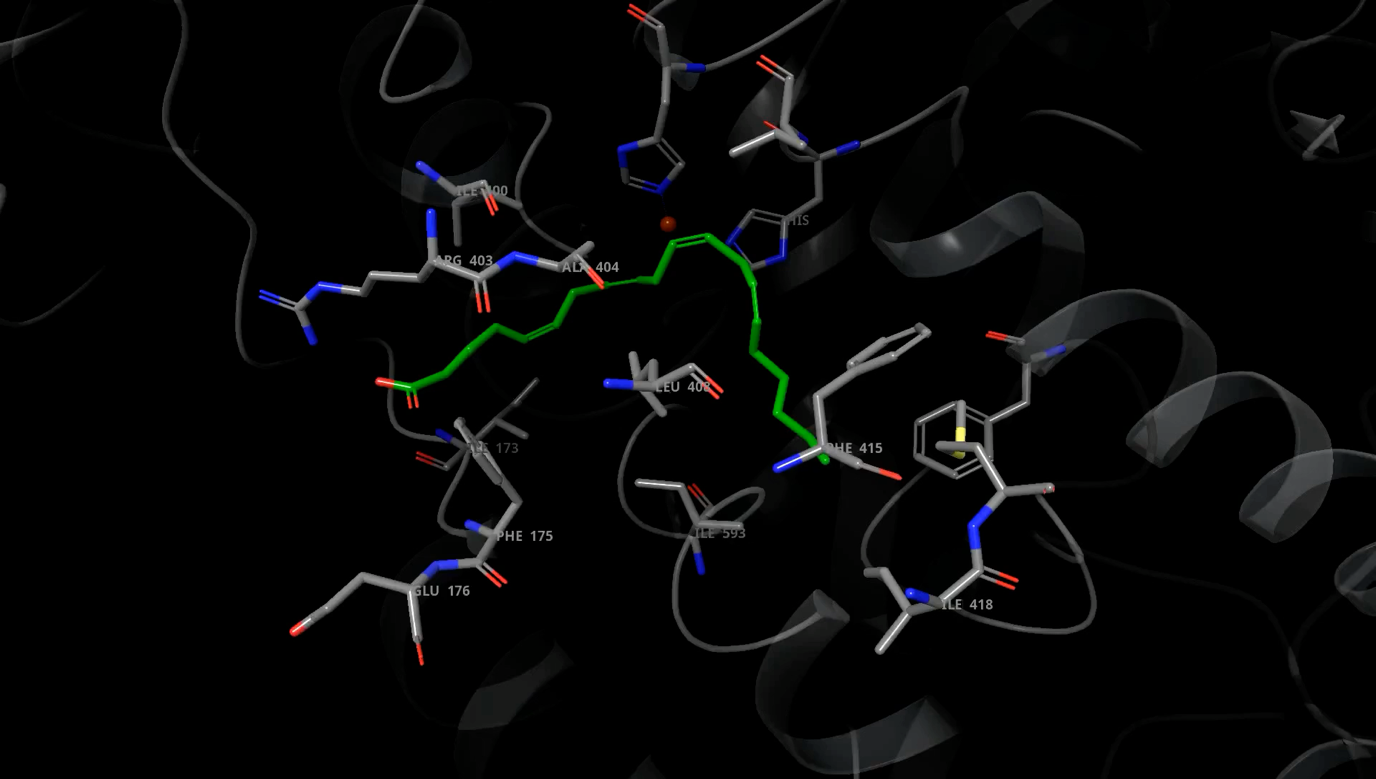
**

**
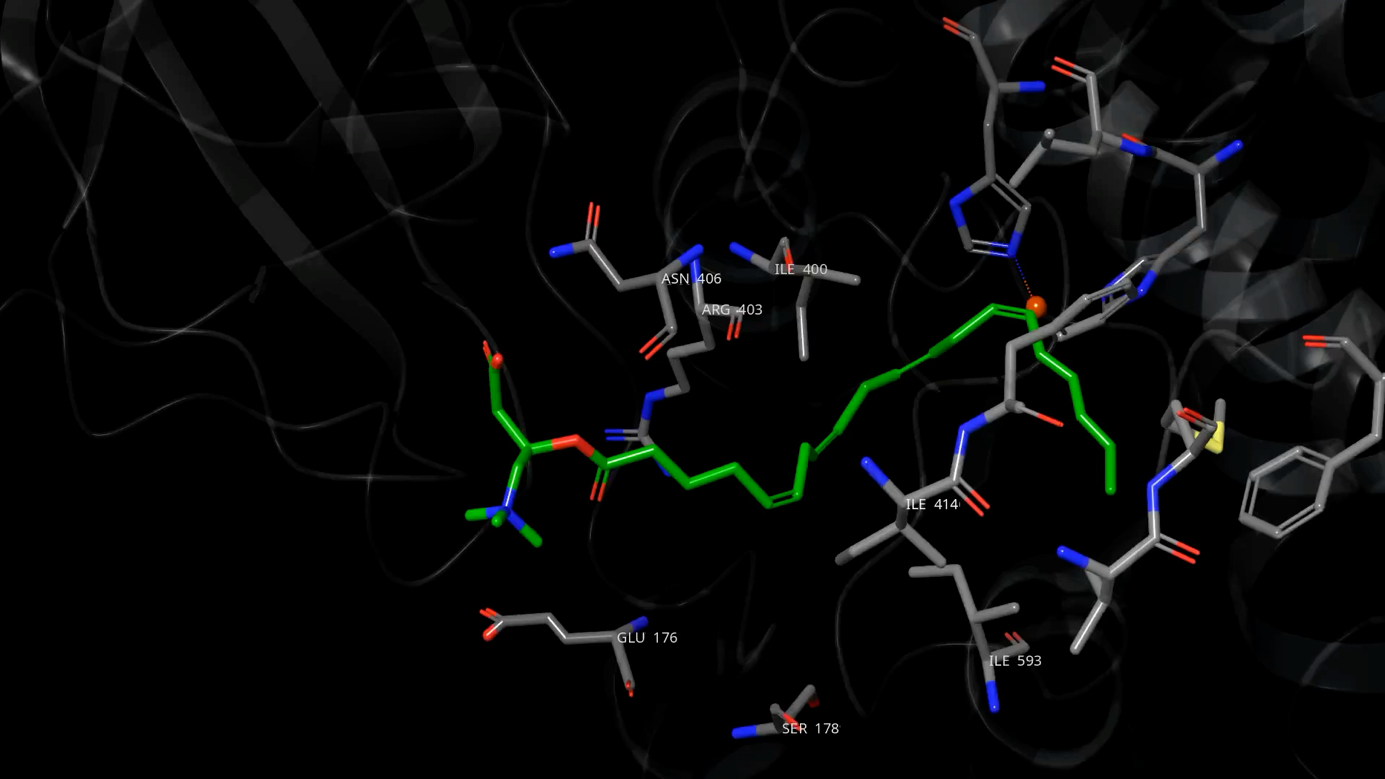
Supplemental movie M2: MD simulation of AA-Car binding at the active site of rabbit ALOX15.** In silico docking studies on the binding of AA-Car at the active site of rabbit ALOX15 and MD-simulations were carried out as described in the Materials and Methods section. A 100 ns simulation period is shown.

**Supplemental movie M3: MD simulation of AA-CoA binding at the active site of rabbit ALOX15.** In silico docking studies on the binding of AA-CoA at the active site of rabbit ALOX15 and MD-simulations were carried out as described in the Materials and Methods section. A 100 ns simulation period is shown.

**
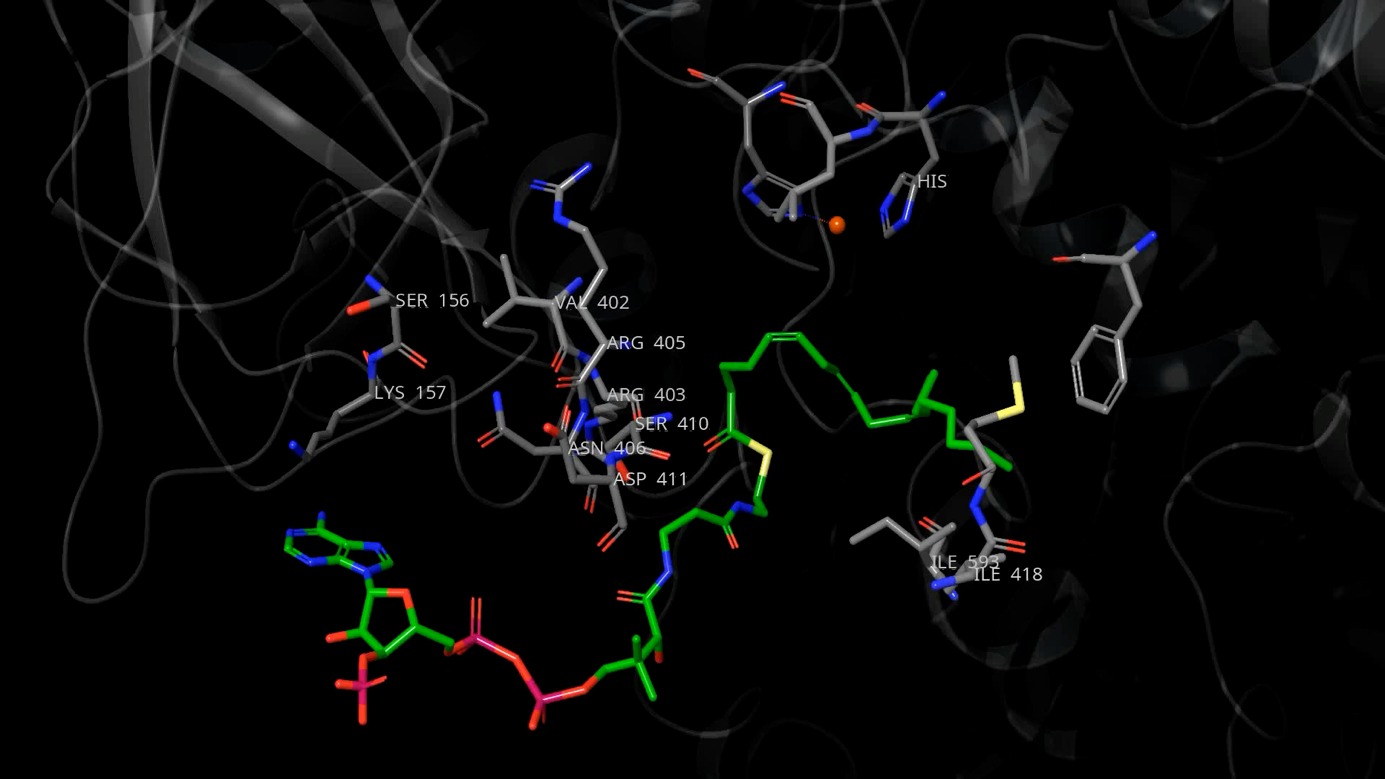
**
